# Integrated Molecular Profiling of Endometrial Cancer: NGS Findings, MSI/MMR Status, Histopathological Features, and TCGA-Inspired Molecular Classification

**DOI:** 10.3390/biomedicines14091880

**Published:** 2026-08-23

**Authors:** Merve Cirak-Balta, Suleyman Erdogdu, Busra Ekinci, Seda Orenay-Boyacioglu, Olcay Boyacioglu, Ibrahim Halil Erdogdu, Nil Culhaci

**Affiliations:** 1 Department of Medical Pathology, School of Medicine, Aydin Adnan Menderes University, Efeler 09010, Aydin, Türkiye; merve.cirak.balta@adu.edu.tr (M.C.-B.); busra.ekinci@adu.edu.tr (B.E.); ibrahim.halil.erdogdu@adu.edu.tr (I.H.E.); nculhaci@adu.edu.tr (N.C.); 2 Department of Medical Oncology, Aydin City Hospital, Efeler 09020, Aydin, Türkiye; dr_suleyman21@hotmail.com; 3 Department of Medical Genetics, School of Medicine, Aydin Adnan Menderes University, Efeler 09010, Aydin, Türkiye; 4 Faculty of Engineering, Aydin Adnan Menderes University, Efeler 09010, Aydin, Türkiye; oboyaci@adu.edu.tr

**Keywords:** endometrial cancer, next-generation sequencing, TCGA molecular classification, microsatellite instability, co-mutation analysis, somatic variations

## Abstract

**Background/Objectives:** Endometrial cancer is a heterogeneous malignancy that displays distinct molecular features and clinical behaviors. The molecular profile of endometrial cancer and its relationship with menopausal status, histological subtype, tumor grade, mismatch repair (MMR)/microsatellite instability (MSI) status, co-mutation patterns, and TCGA molecular classification were investigated in the current study. **Methods:** A total of 81 histopathologically confirmed endometrial cancer cases were retrospectively evaluated. Molecular profiling was performed using a targeted 71-gene NGS panel together with MSI-PCR and mismatch repair (MMR) immunohistochemistry. Because the targeted panel was not designed to detect genome-wide copy-number alterations or comprehensive mutational signatures, molecular subgroup assignment was performed using a surrogate TCGA-inspired molecular classification approach based on *POLE* mutation status, microsatellite instability (MSI)/Mismatch Repair (MMR) findings, and *TP53* alterations. Histological subtypes, tumor grades, menopausal status, and molecular alterations were compared. **Results:** Of the cases evaluated, 57 (70.4%) were endometrioid and 24 (29.6%) were serous carcinoma. *TP53* variations were significantly higher in serous carcinomas compared to endometrioid tumors (66.7% vs. 26.3%; *p* = 0.001). In endometrioid carcinomas, the mean number of variants increased with advancing tumor grade (Grade 1: 4.44; Grade 2: 9.68; Grade 3: 11.42; *p* = 0.006). Alterations in *BLM*, *CDC27*, *MLH3*, and *MSH3* were more frequently observed in high-grade tumors. In exploratory analyses, no significant differences were detected between the premenopausal and postmenopausal groups regarding total variant load, number of altered genes, MSI status, or the distribution of TCGA-inspired molecular subgroups; however, these comparisons were limited by the small number of premenopausal patients. The most frequent co-mutation was observed between *BLM* and *CDC27* (55.6%). In the TCGA-inspired molecular classification, the most prevalent subgroup was NSMP-proxy (33.3%). **Conclusions:** Serous and endometrioid endometrial cancers display distinct molecular characteristics. In endometrioid carcinomas, the genomic alteration load increases in parallel with advancing tumor grade. TCGA-inspired molecular classification and co-mutation analyses revealed the prominent molecular heterogeneity of endometrial cancer. In exploratory analyses, no significant differences were detected between the premenopausal and postmenopausal groups regarding total variant load, number of altered genes, MSI status, or the distribution of TCGA-inspired molecular subgroups. However, these findings should be interpreted with caution because of the limited number of premenopausal patients. Given the limited number of premenopausal patients, findings related to menopausal status should be considered exploratory and require validation in larger, independent cohorts.

## 1. Introduction

Globally, endometrial cancer accounts for approximately 7% of all malignancies and represents the fourth most common cancer in women [1]. In Turkey, the age-standardized incidence rate was reported as 11.3 per 100,000 in 2020, ranking fifth among all cancers [2]. Notably, it is the sole malignancy that has displayed declining survival rates over the last four decades. Mortality rates remain substantially elevated in advanced-stage and high-histological-grade tumor types [3]. Obesity, metabolic syndrome, diabetes mellitus, increased life expectancy, and an aging population are widely accepted as the primary factors driving this escalation. While the prognosis is favorable for a significant proportion of cases diagnosed at an early stage, certain patients display an unexpectedly aggressive clinical course, demonstrating that classifications relying solely on histopathological evaluation have several limitations [4].

For decades, endometrial cancers have been classified into Type I and Type II tumors according to the dualistic model defined by Bokhman. Within this framework, endometrioid tumors are generally considered estrogen-related and low-grade neoplasms with favorable prognoses. Conversely, serous tumors are aggressive, high-grade malignancies with poor outcomes. However, tumors with identical histology often follow markedly different clinical trajectories, highlighting the biological heterogeneity of endometrial cancer and the need for molecular classification [5].

The genomic classification established by The Cancer Genome Atlas (TCGA) project represents a landmark milestone in the molecular-based evaluation of endometrial cancer. Within this framework, tumors are categorized into four distinct molecular subgroups: *POLE*-ultramutated, mismatch repair-deficient/microsatellite instability-high (MMRd/MSI-H), copy-number low (NSMP), and copy-number high/p53-abnormal. Subsequent studies have demonstrated that these molecular subgroups display profound differences not only in terms of genomic features but also regarding prognosis, recurrence risk, and therapeutic response [6,7].

The increasing clinical significance of TCGA-inspired molecular classification has resulted in the development of algorithms such as ProMisE (Proactive Molecular Risk Classifier for Endometrial Cancer) aimed at adapting this approach into routine pathology practice. Currently, molecular classification utilizing *POLE* mutation, MMR protein deficiency, and p53 status has emerged as a critical component of risk assessment in endometrial cancers. Indeed, molecular classification was formally incorporated for the first time into the staging system updated by the International Federation of Obstetrics and Gynecology (FIGO) in 2023, underscoring the pivotal role of molecular data in guiding patient management [8].

Advances in next-generation sequencing (NGS) technologies have enabled a more detailed examination of the molecular architecture of endometrial cancer. In addition to well-known driver genes such as *TP53*, *PTEN*, *PIK3CA*, *KRAS*, *CTNNB1*, and *POLE*, less-studied genomic alterations and co-mutation patterns can be identified. Notably, NGS-based analyses are particularly beneficial for identifying interacting molecular pathways during tumor progression and uncovering novel biomarkers [9].

While numerous studies have addressed the molecular classification of endometrial cancer, comprehensive evaluations that simultaneously assess menopausal status, histological subtype, tumor grade, co-mutation patterns, and TCGA-inspired molecular subgroups within the same patient cohort remain limited. Furthermore, studies establishing the relationship between histopathological features and molecular data derived from targeted NGS panels utilized in routine clinical practice are relatively scarce [5].

The objective of this study is to evaluate next-generation sequencing data from cases diagnosed with endometrial cancer. Molecular variations were investigated within the framework of menopausal status, histological subtype, tumor grade, mismatch repair/MSI status, co-mutation patterns, and TCGA-inspired molecular classification, providing novel data regarding the molecular heterogeneity of endometrial cancer. Accordingly, the current study applied a surrogate TCGA-inspired molecular classification based on targeted sequencing, MSI-PCR, and MMR immunohistochemistry.

## 2. Materials and Methods

### 2.1. Ethical Approval and Study Population

This study was approved by the Non-Interventional Clinical Research Ethics Committee of Aydin Adnan Menderes University School of Medicine (Decision No: 2025/#119) and was conducted in accordance with the principles of the Declaration of Helsinki.

The study was retrospectively designed, and the records of endometrial cancer patients followed at the Oncology Clinic of Aydin Adnan Menderes University School of Medicine and referred to the Molecular Pathology Laboratory between 2020 and 2025 were reviewed. Patients with histopathologically confirmed diagnoses who underwent NGS gene mutation panel analysis and possessed complete MSI status data were included in the study. Conversely, cases with missing clinical or molecular testing data were excluded.

A total of 81 patients meeting the designated criteria were included in the analyses. For each patient, age, tumor histology, immunohistochemical and MSI data, and somatic mutation profiles were evaluated. In cases where direct clinical information was unavailable, menopausal status was defined based on age. Accordingly, patients were categorized into two groups for evaluation: the premenopausal group aged 18–49 years and the postmenopausal group aged 50 years and older. MSI analyses and NGS studies were performed in accordance with the standard operating procedures of the laboratory.

### 2.2. Microsatellite Instability (MSI) Analysis

The MSI status was evaluated using both molecular methods and immunohistochemical examinations, in accordance with the procedures described by Taskiran et al. (2025) and Erdogdu et al. (2024) [10,11].

For MSI analysis, the EasyPGX^®^ Ready MSI kit (Diatech Pharmacogenetics, Ancona, Italy) was utilized. The BAT-25, BAT-26, NR-21, NR-24, and MONO-27 loci were examined in DNA samples obtained from tumor tissues. As the primer sequences used were not disclosed by the manufacturer, they were not specified in this study. Cases displaying instability at a single locus were evaluated as MSI-low (MSI-L), whereas the presence of instability at two or more loci was considered MSI-high (MSI-H). Samples demonstrating stability across all examined loci were categorized into the microsatellite stable (MSS) group.

MMR protein expressions were investigated via immunohistochemical methods. Sections prepared from formalin-fixed, paraffin-embedded (FFPE) tissue blocks were stained utilizing standard laboratory protocols. The MLH1, PMS2, MSH2, and MSH6 proteins were evaluated. Staining procedures were performed on an automated staining platform, and the immunoreactions were visualized using diaminobenzidine.

During evaluation, the presence of nuclear staining within tumor cells was examined. Cases demonstrating a loss of expression in any of the MLH1, PMS2, MSH2, or MSH6 proteins were classified as dMMR. Conversely, the retention of nuclear expression across all four proteins was evaluated as proficient mismatch repair (pMMR).

To establish the final MSI classification, PCR results and immunohistochemical findings were evaluated in combination.

### 2.3. Next-Generation Sequencing (NGS) Analysis

For molecular analyses, tumor tissues obtained from the FFPE blocks were utilized. Genomic DNA isolation was performed using the QIAamp DNA FFPE Tissue Kit (Qiagen, Hilden, Germany) in accordance with the manufacturer’s recommendations. DNA quality and quantity measurements were evaluated spectrophotometrically; samples with an OD_260_/OD_280_ ratio between 1.8 and 2.0 were considered suitable for analysis.

Following library preparation, sequencing was performed on the Illumina MiSeq platform (MiniSEQ, MN00676, Illumina, Hayward, CA, USA) as previously described by Erdogdu et al. (2024) and Cokpinar et al. (2024) [11,12]. The QIAseq Human Solid Organ Cancer Panel (Qiagen, Hilden, Germany), which covered 71 genes associated with endometrial cancer, was utilized for the analyses (*POLE*, *CDC27*, *BLM*, *PTEN*, *TP53*, *MLH1*, *MLH3*, *MSH2*, *MSH3*, *MSH6*, *PMS1*, *PMS2*, *PIK3CA*, *ATM*, *CHEK2*, *ERBB2*, *FGFR3*, *TGFBR2*, *PIK3R1*, *BRCA1*, *BRCA2*, *ACVR1B*, *RET*, *MET*, *EGFR*, *APC*, *KRAS*, *NRAS*, *BRAF*, *ATR, KMT2C*, *STK11*, *AXIN2*, *SMAD2*, *SMAD4*, *BAX*, *BUB1B*, *CDH1*, *CDK4*, *CDKN2A*, *CTNNA1*, *PTPN12*, *DCC*, *WBSCR17*, *ENG*, *FBXW7*, *FZD3*, *GPC3*, *MAP2K4*, *MAP7*, *MYO1B*, *RPS20*, *SLC9A9*, *SRC*, *MIER3*, *TCERG1*, *TCF7L2*, *CASP8*, *CTNNB1*, *EP300*, *EPCAM*, *GREM1*, *BMPR1A*, *MUTYH*, *DMD*, *GALNT12*, *ATP6V0D2*, *POLD1*, *SCG5*, *FLCN*, *AKT1*). The panel encompassed all exonic regions and exon-intron boundaries of the targeted genes.

The mean sequencing depth across targeted regions was greater than 500×, and only variants with a coverage depth of at least 100× were considered for evaluation. Raw data were analyzed using the Qiagen Clinical Insight Interpret (QCI^TM^) software (QCI Interpret v2.1, accessed between 2020 and 2025; QIAGEN Digital Insights, Redwood City, CA, USA). To ensure reliable identification of somatic variants, a threshold of 5% was applied for variant allele frequency (VAF). Variants that failed to meet the predefined quality control criteria, including a minimum coverage depth of 100× and a VAF of at least 5%, were considered potential sequencing artifacts and excluded from downstream analyses.

Detected variants were classified according to the American College of Medical Genetics and Genomics (ACMG) guidelines. Variants evaluated as pathogenic and likely pathogenic were included in the statistical analyses. Although variants of uncertain significance (VUS) were recorded, they were excluded from comparative analyses as their biological and clinical impacts could not be clearly established. Benign and likely benign variants were excluded from evaluation.

#### TCGA-Inspired Molecular Classification

Because the QIAseq Human Solid Organ Cancer Panel (Qiagen, Hilden, Germany) interrogated only predefined coding regions, it could not reliably detect genome-wide copy-number alterations or comprehensive mutational signatures. Therefore, molecular subgroup assignment was performed using a surrogate TCGA-inspired molecular classification rather than the original TCGA genomic classification.

- Tumors harboring pathogenic or likely pathogenic *POLE* variants were classified as the *POLE*-mutated proxy subgroup within the TCGA-inspired molecular classification framework. *POLE* variants were subsequently categorized as Exonuclease Domain Mutation (EDM) hotspot, EDM, or Non-EDM.
- Tumors demonstrating mismatch repair deficiency by immunohistochemistry and/or MSI-high status by PCR were classified as the MMRd/MSI-H subgroup.
- Tumors harboring pathogenic *TP53* alterations detected by targeted sequencing were classified as the p53-abnormal proxy subgroup.
- Because p53 immunohistochemistry was not incorporated into the molecular subgroup assignment, pathogenic *TP53* variants identified by targeted sequencing were used as a surrogate marker of the p53-abnormal subgroup.
- Remaining tumors were classified as the No Specific Molecular Profile (NSMP)-proxy subgroup.

Because individual tumors could harbor more than one molecular classifier, a hierarchical classification algorithm was applied. Tumors carrying pathogenic or likely pathogenic *POLE* variants were assigned to the *POLE*-mutated proxy subgroup irrespective of additional alterations. Remaining tumors demonstrating MMR deficiency and/or MSI-H status were assigned to the MMRd/MSI-H subgroup. Tumors not classified as *POLE*-mutated proxy or MMRd/MSI-H but harboring pathogenic *TP53* alterations were assigned to the p53-abnormal subgroup. All remaining tumors were classified as NSMP. The overlap between molecular classifiers before application of the hierarchical algorithm is presented in Supplementary Table S3.

### 2.4. Statistical Analysis

**Figure 1:**
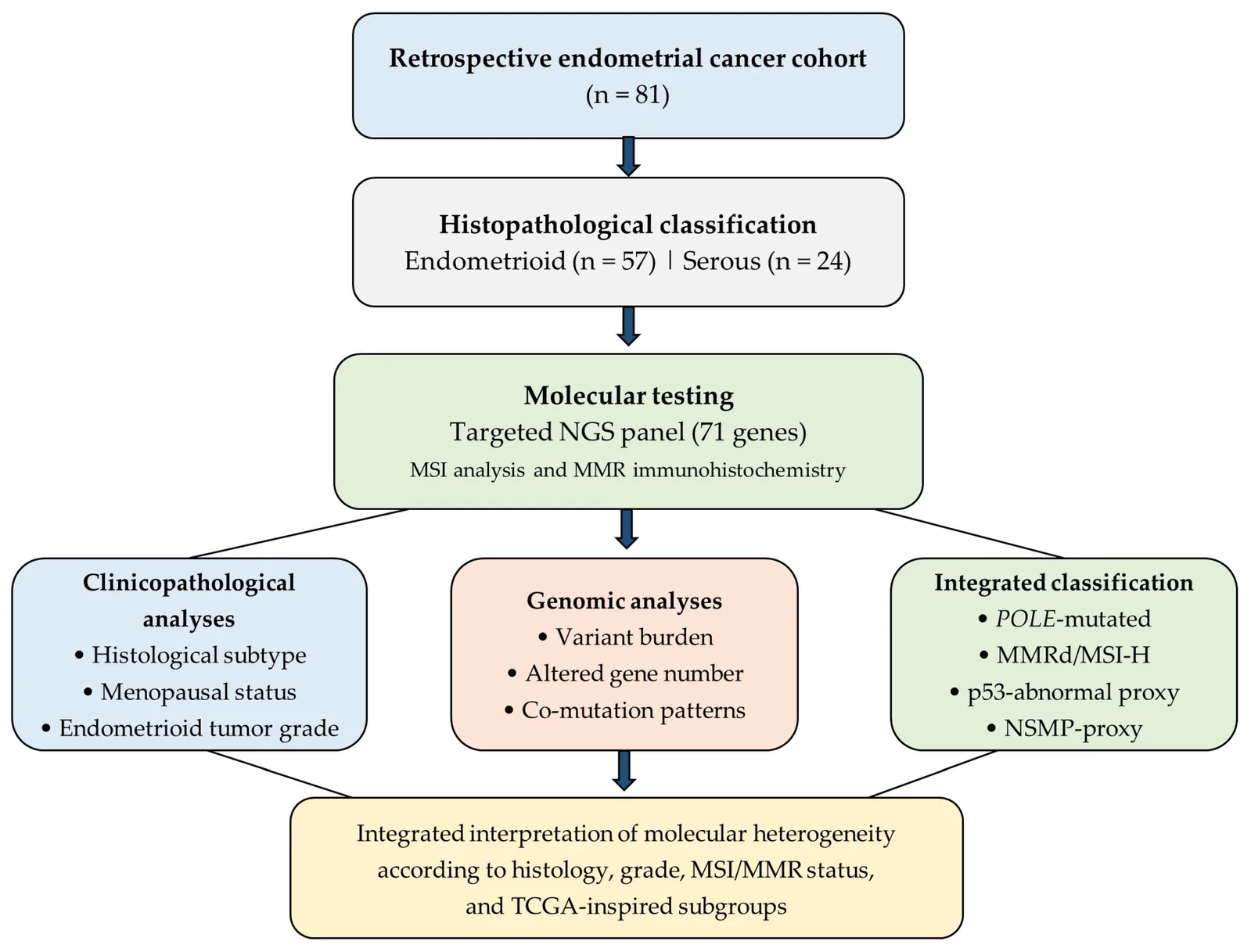
Study Workflow. MMR: mismatch repair; MSI: microsatellite instability; NGS: next-generation sequencing; NSMP: no specific molecular profile. Molecular subgroup assignment was based on pathogenic *POLE* EDM hotspot, EDM, Non-EDM variants, MSI-PCR/MMR immunohistochemistry, *TP53* pathogenic variants, and absence of these alterations (NSMP-proxy).

Statistical analyses were performed using IBM SPSS Statistics version 25.0 (IBM Corp., Armonk, NY, USA). Continuous variables were presented as mean ± standard deviation (SD) or median (minimum–maximum), whereas categorical variables were expressed as frequencies and percentages. Comparisons between two categorical variables were performed using Pearson’s chi-square test or Fisher’s exact test, as appropriate according to the expected cell counts. Comparisons of continuous variables among three groups were performed using one-way analysis of variance (ANOVA), whereas categorical variables involving more than two groups were analyzed using the Pearson chi-square test or the Fisher–Freeman–Halton exact test, as appropriate. Co-mutation frequencies were compared using Pearson’s chi-square test or Fisher’s exact test according to the expected cell counts. To control for multiple testing, the Benjamini–Hochberg false discovery rate (BH-FDR) correction was applied separately to each predefined family of comparisons (histological subtype, menopausal status, endometrioid tumor grade, and co-mutation analyses). For co-mutation analyses, BH-FDR correction was applied across 11 pairwise comparisons. Both raw *p* values and adjusted q values are reported, and q < 0.05 was considered statistically significant. The study workflow is summarized in Figure 1.

## 3. Results

### 3.1. Clinicopathological Characteristics of the Cohort

A total of 81 endometrial cancer cases were evaluated in this study. Regarding histological subtypes, 57 cases (70.4%) were classified as endometrioid carcinoma, whereas 24 cases (29.6%) were categorized as serous carcinoma. When endometrioid carcinomas were evaluated according to tumor grade, Grade 1, Grade 2, and Grade 3 tumors accounted for 15.8%, 59.6%, and 24.6% of the cases, respectively. In terms of menopausal status, 8 patients (9.9%) were premenopausal and 73 patients (90.1%) were postmenopausal. The mean age was found to be 44.9 ± 5.5 years in the premenopausal group and 65.3 ± 7.4 years in the postmenopausal group.

A total of 756 variant records were examined across the entire cohort. The mean number of variants per case was 9.12 ± 5.34 in endometrioid carcinomas and 9.83 ± 6.68 in serous carcinomas (*p* = 0.284). Similarly, the mean number of mutated genes per case was calculated as 6.79 ± 4.01 in endometrioid carcinomas and 7.88 ± 4.86 in serous carcinomas (*p* = 0.331).

When endometrioid carcinomas were evaluated according to tumor grade, the mean number of variants was found to be 4.44 ± 2.19 in Grade 1 tumors, 9.68 ± 5.37 in Grade 2 tumors, and 11.42 ± 6.11 in Grade 3 tumors (*p* = 0.006). The number of mutated genes per case was 3.78 ± 1.39, 7.41 ± 3.84, and 8.17 ± 4.72, respectively (*p* = 0.021). No significant difference was observed between the histological subtypes in terms of total variant load and the number of mutated genes per case.

Regarding menopausal status, the number of variants per case was 8.75 ± 4.92 in the premenopausal group and 9.40 ± 5.84 in the postmenopausal group. Similarly, the number of mutated genes per case was found to be 6.00 ± 3.02 in the premenopausal group and 7.11 ± 4.29 in the postmenopausal group. No significant differences were detected between the two groups based on menopausal status regarding total variant load and the number of mutated genes per case (*p* = 0.726 and *p* = 0.479, respectively).

In our molecular classification analysis, the NSMP-proxy subgroup was identified as the most frequent category, which was followed by the *POLE*-mutated proxy, p53-abnormal proxy, and MMRd/MSI-H subgroups. In the MMR/MSI evaluation, the loss of MLH1 and PMS2 expressions along with the MMRd/MSI-H phenotype was observed in a distinct portion of the cohort, a finding consistent with the molecular heterogeneity of endometrial cancer. The clinicopathological and molecular characteristics of the patients are summarized in Table 1.

### 3.2. Histological Subtype-Based Molecular Findings

In endometrioid carcinomas, *BLM* variations were most frequently identified (77.2%), which were followed by *CDC27* (57.9%), *MLH3* (52.6%), *MSH3* (45.6%), *PTEN* (43.9%), *POLE* (31.6%), *PIK3CA* (29.8%), and *TP53* (26.3%). In serous carcinomas, *BLM* (87.5%), *CDC27* (70.8%), *TP53* (66.7%), *PIK3CA* (50.0%), *MSH3* (41.7%), *PTEN* (33.3%), *POLE* (33.3%), and *MLH3* (29.2%) variations were prominent.

*TP53* alterations remained significantly more frequent in serous carcinomas than in endometrioid carcinomas after BH-FDR correction (raw *p* = 0.001, BH-FDR q = 0.015). In contrast, the nominal associations observed for *CTNNB1* and *KRAS* did not remain statistically significant after multiple-testing correction (both q = 0.140). No significant differences were observed between the two histological subtypes regarding *PTEN*, *PIK3CA*, *POLE*, *BLM*, *CDC27*, *MLH3*, and *MSH3* variations.

Detailed information on all unique pathogenic and likely pathogenic variants identified in the study cohort is presented in Supplementary Table S1. A complete oncoprint including all 81 endometrial cancer cases and all pathogenic or likely pathogenic alterations identified in the study cohort is provided in Supplementary Figure S1.

**Figure 2:**
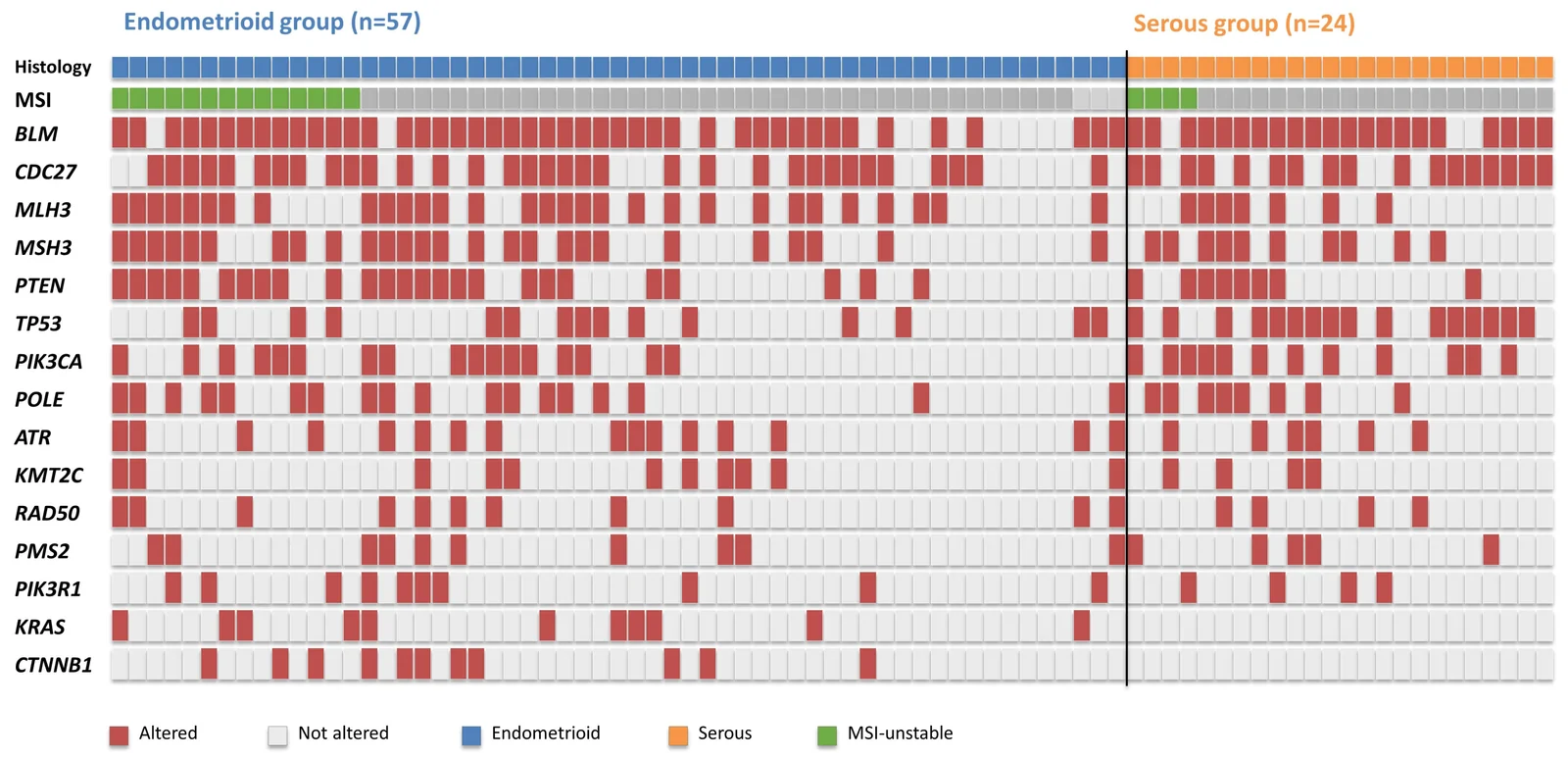
Oncoprint of major molecular alterations in the study cohort.

When evaluated in terms of MMR/MSI status, MLH1 loss, PMS2 loss, and MSI were detected in 24.6%, 22.8%, and 24.6% of endometrioid carcinomas, respectively. In serous carcinomas, MSI instability was observed at a rate of 16.7%. Although the MMRd/MSI-H rate appeared higher in endometrioid tumors, this difference was not statistically significant (*p* = 0.564) (Table 2 and Figure 2).

### 3.3. Molecular Alterations According to Menopausal Status

When evaluated at the gene level, *BLM* variations were most frequently observed in the premenopausal group and were detected in all cases (8/8, 100%). This was followed by *CDC27* (62.5%), *MLH3* (50.0%), *ATR* (37.5%), *MSH3*, *PTEN*, *POLE*, *CTNNB1*, and *KMT2C* (each 25.0%). In the postmenopausal group, however, *BLM* variations were identified in 57 cases (78.1%), *CDC27* in 45 cases (61.6%), *MSH3* in 34 cases (46.6%), *MLH3* in 33 cases (45.2%), *PTEN* in 31 cases (42.5%), *TP53* in 30 cases (41.1%), *PIK3CA* in 28 cases (38.4%), and *POLE* in 24 cases (32.9%).

When comparing gene frequencies, no statistically significant differences were observed between the groups. Nevertheless, *TP53* and *PIK3CA* variations were found to occur at higher rates in the postmenopausal group. Specifically, *TP53* variations were detected in 12.5% of the premenopausal group and 41.1% of the postmenopausal group (*p* = 0.145), while *PIK3CA* variations were observed at rates of 12.5% and 38.4%, respectively (*p* = 0.248).

Upon evaluating MMR immunohistochemistry and MSI findings, MLH1 loss was identified in 2 cases (25.0%) in the premenopausal group and 16 cases (21.9%) in the postmenopausal group (*p* = 1.000). Similarly, PMS2 loss was observed at rates of 25.0% and 20.5%, respectively (*p* = 0.672). In contrast, no loss of MSH2 or MSH6 expression was observed in the premenopausal group. MSI was detected in 25.0% of premenopausal cases and 21.9% of postmenopausal cases, demonstrating no significant difference between the groups (*p* = 1.000). After BH-FDR correction, no gene-level associations remained statistically significant. Given the small number of premenopausal patients (*n* = 8), these subgroup comparisons should be regarded as exploratory and interpreted with caution.

### 3.4. Grade-Specific Molecular Alterations in Endometrioid Carcinomas

When endometrioid carcinomas were evaluated according to tumor grade, an increase in genomic alteration burden was observed with increasing grade. The mean number of variants was found to be 4.44 in Grade 1 tumors, 9.68 in Grade 2 tumors, and 11.42 in Grade 3 tumors (*p* = 0.006). Similarly, a significant increase was detected in the number of mutated genes per case (*p* = 0.021).

*BLM* variations were observed at rates of 33.3%, 85.3%, and 83.3% in Grade 1, Grade 2, and Grade 3 tumors, respectively (*p* = 0.004). *CDC27* variations were found at rates of 22.2%, 64.7%, and 75.0%, respectively (*p* = 0.034). While *MLH3* variations were detected in 11.1%, 64.7%, and 58.3% of the cases (*p* = 0.016), *MSH3* variations were observed at rates of 11.1%, 50.0%, and 66.7%, in the same order (*p* = 0.036). After BH-FDR correction, only the association between *BLM* alterations and tumor grade remained statistically significant (BH-FDR q = 0.030). The nominal associations observed for *CDC27*, *MLH3*, and *MSH3* did not retain statistical significance after multiple-testing correction (q > 0.05).

Although *PTEN* and *PIK3CA* variations were observed to occur more frequently in high-grade tumors, these differences did not reach statistical significance (*p* = 0.125 and *p* = 0.154, respectively). In contrast, *TP53* variations were found not to be associated with histological grade in endometrioid tumors (*p* = 0.901).

The MMRd/MSI-H rate was found to be 11.1% in Grade 1 tumors, 26.5% in Grade 2 tumors, and 28.6% in Grade 3 tumors. Although a trend toward an increase in the MMRd/MSI-H rate was observed with increasing grade, the difference between the groups was not statistically significant (*p* = 0.417) (Table 3).

### 3.5. Co-Mutation Analysis

Co-mutation analysis demonstrated the presence of a BLM-centered co-occurrence pattern across the entire cohort. The most frequent co-occurrence was observed between *BLM* and *CDC27*, which was detected in 45 cases (55.6%). This was followed by *BLM–MLH3* (42.0%), *BLM–MSH3* (40.7%), *CDC27–MSH3* (37.0%), *CDC27–MLH3* (35.8%), *BLM–PTEN* (34.6%), and *MLH3–MSH3* (33.3%) co-occurrences.

In endometrioid carcinomas, the most frequent co-mutations were *BLM–CDC27* (52.6%), *BLM–MLH3* (47.4%), *BLM–MSH3* (42.1%), *CDC27–MLH3* (42.1%), and *CDC27–MSH3* (38.6%). In serous carcinomas, *BLM–CDC27* (62.5%), *BLM–TP53* (54.2%), *CDC27–TP53* (45.8%), *TP53–PIK3CA* (41.7%), and *BLM–PIK3CA* (37.5%) were the most frequent co-mutation pairs.

Histology-specific comparisons showed that *BLM–TP53* co-mutation was significantly more frequent in serous carcinomas than in endometrioid carcinomas (54.2% vs. 22.8%, raw *p* = 0.009). Similarly, *TP53–PIK3CA* (41.7% vs. 10.5%, raw *p* = 0.004) and *CDC27–TP53* (45.8% vs. 17.5%, raw *p* = 0.012) co-mutations were enriched in serous carcinomas.

In endometrioid carcinomas, an increase in the number of co-mutations was observed with increasing tumor grade. While *BLM–CDC27* was the dominant co-mutation pair in Grade 1 tumors, *BLM–MLH3*, *BLM–MSH3*, and *CDC27–MSH3* became more prominent in Grade 2 tumors. In Grade 3 tumors, *BLM–PTEN, BLM–PIK3CA*, and *MLH3–MSH3* combinations appeared relatively more frequent. Concurrent alterations involving DNA repair genes and the PI3K/PTEN pathway were particularly noticeable in higher-grade tumors.

Following BH-FDR correction applied across the 11 predefined co-mutation comparisons presented in Table 4, *BLM–TP53*, *TP53–PIK3CA*, and *CDC27–TP53* remained statistically significant (all adjusted q = 0.044), whereas the remaining co-mutation pairs did not retain statistical significance after multiple-testing correction.

### 3.6. TCGA-Inspired Molecular Classification Results

In the TCGA-inspired molecular classification, the NSMP-proxy subgroup was identified as the most frequent category across the entire cohort (34.6%). This was followed by the *POLE*-mutated proxy (32.1%), p53-abnormal proxy (21.0%), and MMRd/MSI-H (12.3%) groups.

When evaluated in terms of histological subtypes, the NSMP-proxy group was dominant in endometrioid carcinomas (40.4%). Within the endometrioid cases, 31.6% fell into the *POLE*-mutated proxy group, 15.8% into the p53-abnormal proxy group, and 12.3% into the MMRd/MSI-H group. In contrast, the p53-abnormal proxy group was observed to be represented at a prominently higher rate in serous carcinomas (41.7%). In these serous cases, the *POLE*-mutated, NSMP-proxy, and MMRd/MSI-H groups were detected at rates of 33.3%, 16.7%, and 8.3%, respectively.

When endometrioid carcinomas were examined according to tumor grade, the vast majority of Grade 1 tumors fell into the NSMP-proxy group (66.7%). In Grade 2 tumors, the NSMP-proxy and POLE-mutated groups were represented at similar rates. In Grade 3 tumors, however, a relative increase was observed in the p53-abnormal proxy and MMRd/MSI-H subgroups. The distribution of TCGA- inspired molecular subgroups differed significantly between endometrioid and serous carcinomas (Pear-son’s χ^2^ test, *p* = 0.012, q = 0.012), with p53-abnormal tumors being more frequent in serous carcinomas and NSMP tumors being more frequent in endometrioid carcino-mas. No statistically significant difference was observed in the distribution of TCGA-inspired molecular subgroups across endometrioid tumor grades (raw *p* = 0.084, BH-FDR q = 0.084). (Table 5). 

Detailed *POLE* variant characteristics are provided in Supplementary Table S2, whereas variant-level information for *POLE*, *TP53*, *BLM*, *CDC27*, *MLH3*, and *MSH3*, including VAF and sequencing coverage values, is presented in Supplementary Table S3.

The overlap between molecular classifiers before hierarchical subgroup assignment is presented in Supplementary Table S4.

The mean variant burden according to the TCGA-inspired molecular subgroups is summarized in Supplementary Table S5.

## 4. Discussion

In this study, next-generation sequencing data from 81 endometrial cancer cases were evaluated within the framework of histological subtype, endometrioid tumor grade, menopausal status, co-mutation patterns, and TCGA-inspired molecular classification. Our findings demonstrated that there was no prominent molecular segregation according to menopausal status; in contrast, more significant molecular differences emerged regarding histological subtype and tumor grade. Particularly, the marked elevation of *TP53* variations in serous carcinomas, the presence of *CTNNB1* and *KRAS* alterations exclusively within the endometrioid group, and the increase in variant burden with increasing grade in endometrioid tumors appeared consistent with current molecular classification approaches. Accordingly, the molecular subgroups reported in this study represent surrogate categories derived from targeted sequencing, MSI-PCR, and MMR immunohistochemistry rather than the original TCGA genomic framework.

The most significant shift in endometrial cancer management over the past decade has been the integration of molecular classification systems into traditional histopathological classification. The original groups defined by the TCGA have subsequently been adopted in clinical practice as the *POLE*-mutated proxy, MMRd, p53-abnormal, and NSMP subgroups, which offer enhanced clinical applicability. The ProMisE approach has also integrated this molecular classification into the practical pathology workflow, significantly increasing the importance of *POLE* mutation, MMR protein loss, and p53 status in risk stratification [7,13]. Indeed, the inclusion of molecular subgroups in staging and risk assessment processes in the 2023 FIGO update clearly demonstrates that this approach has become a standard component in management strategies [7,8,13].

In the current cohort, the identification of the NSMP-proxy and *POLE*-mutated proxy groups as the most frequent molecular categories further supports the molecular heterogeneity of endometrial cancer. However, the molecular subgroup assignment in the present study was based on a targeted 71-gene NGS panel combined with MSI-PCR and MMR immunohistochemistry rather than whole-exome or whole-genome sequencing. Therefore, the molecular classification should be interpreted as a TCGA-inspired classification. Although this integrated approach is practical and applicable in routine clinical practice, the targeted panel is not designed to detect genome-wide copy-number alterations or comprehensive mutational signatures. Therefore, all molecular subgroups identified in this study (*POLE*-mutated proxy, MMRd/MSI-H, p53-abnormal, and NSMP) should be interpreted as surrogate molecular categories rather than direct TCGA molecular subgroups derived from genome-wide genomic profiling.

*TP53* alterations were more frequent in serous carcinomas than in endometrioid carcinomas, and this association remained significant after BH-FDR correction, supporting the well-established role of *TP53* abnormalities in serous endometrial carcinoma. Serous endometrial cancers are classically characterized as high-grade, aggressive tumors that largely correspond to the p53-abnormal/copy-number high molecular subgroup. In current molecular classification studies, the p53-abnormal group is reported to be particularly associated with serous histology and high-grade endometrioid tumors, as well as being linked to poorer clinical outcomes [14,15,16]. In this study, the detection of *TP53* variations at a rate of 66.7% in serous carcinomas and 26.3% in endometrioid carcinomas—with this difference being statistically significant—further supports the p53-dominant molecular profile of serous carcinomas.

*CTNNB1* and *KRAS* alterations were detected exclusively in endometrioid carcinomas. However, these associations did not remain statistically significant after BH-FDR correction for multiple testing and should therefore be interpreted as exploratory findings requiring validation in larger independent cohorts. Nevertheless, the distribution of these alterations is consistent with the established molecular characteristics of endometrioid endometrial carcinoma. *CTNNB1*, a key component of the Wnt/β-catenin signaling pathway, is among the most frequently altered genes in low-grade, early-stage endometrioid tumors and has recently been recognized as an additional prognostic marker within the NSMP molecular subgroup, where it has been associated with an increased risk of recurrence. Likewise, *KRAS* alterations are well-recognized drivers of PI3K/RTK/RAS pathway activation and are predominantly observed in endometrioid carcinomas. Although the associations identified in the present study did not retain statistical significance after multiple-testing correction, the complete absence of *CTNNB1* and *KRAS* alterations in serous carcinomas supports the concept that endometrioid and serous endometrial cancers arise through distinct molecular pathways, consistent with previous genomic and molecular classification studies [17,18].

When MMR/MSI findings were evaluated, the MMRd/MSI-H rate in endometrioid carcinomas appeared higher relative to serous carcinomas; nevertheless, this difference did not reach statistical significance. In endometrial cancers, MMR defect is particularly associated with endometrioid histology, and the MMRd/MSI-H phenotype is reported in nearly one-third of cases across many series. MMRd tumors carry clinical significance both for Lynch syndrome screening and for immunotherapy response [9,19,20]. In this study, the detection of MSI at a rate of 24.6% in endometrioid carcinomas and 16.7% in serous carcinomas is consistent with the direction of the literature, though it did not produce a significant difference due to sample size.

The increase in the mean number of variants and mutated genes with increasing grade in endometrioid carcinomas suggests a potential relationship between tumor progression and genomic complexity. While the mean number of variants was 4.44 in Grade 1 tumors, it increased to 11.42 in Grade 3 tumors. Similarly, the number of mutated genes showed an upward trend from Grade 1 to Grade 3. This finding demonstrates that high-grade endometrioid tumors harbor a more complex structure not only morphologically but also molecularly. It is reported in the literature that the p53-abnormal subgroup and indicators of genomic instability can be observed more frequently in high-grade endometrioid tumors [15,16]. In our data, *TP53* did not show a significant difference among endometrioid grade groups; in contrast, *BLM*, *CDC27*, *MLH3*, and *MSH3* variations demonstrated a marked increase in Grade 2 and Grade 3 tumors.

Among the grade-associated alterations, only the association for *BLM* remained significant after BH-FDR correction, whereas the associations observed for *CDC27*, *MLH3*, and *MSH3* should be regarded as nominal findings that require validation in larger cohorts.

The *BLM* gene is a member of the RecQ helicase family and is involved in DNA replication, homologous recombination, and double-strand break repair. Loss of *BLM* function has been associated with chromosomal instability and cancer susceptibility; furthermore, it was reported that *BLM* expression and alterations might carry significance within the context of genomic instability in various tumor types [21,22]. *CDC27*, on the other hand, is a component of the anaphase-promoting complex/cyclosome structure and plays a role in mitotic progression, chromosome segregation, and cell cycle regulation. The role of *CDC27* in cancer biology is not yet as clearly defined as that of classic driver genes; nevertheless, it was indicated that disruptions in the APC/C complex might be linked to mitotic checkpoint failure and aneuploidy [23,24]. In this study, the observation of the *BLM–CDC27* co-mutation as the most frequent combination across the entire cohort suggests that the cell cycle and genome stability axis becomes prominent in this series.

The increase in *MLH3* and *MSH3* variations, particularly in high-grade endometrioid tumors, is also noteworthy. Although *MLH3* and *MSH3* are not utilized in routine clinical classification as extensively as the classic MMR proteins (*MLH1*, *MSH2*, *MSH6*, and *PMS2*), they function as accessory components of the mismatch repair mechanism. It has been discussed in the literature that *MSH3* alterations in endometrial cancers can be observed particularly in MSI-positive tumors, and in some cases, whether they are the cause or the consequence of MSI remains a subject of debate [25,26]. Similarly, while the role of *MLH3* in endometrial cancer has been investigated, its pathogenic effect and clinical utility are not as clearly established as those of the classic MMR genes [27,28].

In conclusion, the more frequent detection of *BLM*, *CDC27*, *MLH3*, and *MSH3* variations in high-grade tumors in our study further suggests that DNA repair mechanisms and cell cycle control become progressively disrupted during tumor progression. These findings support the premise that, alongside well-defined driver genes such as *TP53*, *PTEN*, and *PIK3CA*, additional molecular pathways involved in maintaining genome integrity may also contribute to the biological heterogeneity of endometrial cancer. Nevertheless, the findings regarding *BLM*, *CDC27*, *MLH3*, and *MSH3* do not possess the same level of clinical establishment as well-known endometrial cancer driver genes like *PTEN*, *PIK3CA*, *ARID1A*, *TP53*, and *CTNNB1*. Therefore, the findings regarding *BLM*, *CDC27*, *MLH3*, and *MSH3* should be regarded as exploratory and hypothesis-generating rather than confirmatory. Their biological and clinical significance requires validation in larger independent cohorts and functional studies before definitive conclusions can be drawn.

In the analysis performed according to menopausal status, no statistically significant differences were detected regarding total variant burden, number of mutated genes, MSI/MMR status, or major gene frequencies. The numerically higher prevalence of *TP53* and *PIK3CA* variations in the postmenopausal group may represent a biologically plausible trend. However, the inclusion of only eight premenopausal patients substantially limits the statistical power of these comparisons. Therefore, all findings related to menopausal status should be considered exploratory and interpreted with caution rather than as evidence of definitive molecular segregation.

Co-mutation analysis revealed distinct co-occurrence patterns across histological subtypes. Co-mutations involving *BLM–CDC27*, *BLM–MLH3*, *BLM–MSH3*, and *CDC27–MLH3* were more frequently observed in endometrioid carcinomas, whereas *BLM–TP53*, *CDC27–TP53*, and *TP53–PIK3CA* co-mutations were more prominent in serous carcinomas. This distribution suggests that genomic instability in serous carcinomas may be driven predominantly by *TP53*-related molecular events, whereas endometrioid carcinomas appear to harbor a more complex co-mutation network involving DNA damage repair and cell-cycle regulatory pathways. Furthermore, the increasing diversity of co-mutation patterns observed with higher tumor grade in endometrioid carcinomas parallels the progressive increase in variant burden identified in these tumors. After BH-FDR correction, *BLM–TP53*, *TP53–PIK3CA*, and *CDC27–TP53* co-mutations remained statistically significant, whereas the remaining co-mutation pairs did not retain statistical significance.

The most significant strength of this study is the comprehensive evaluation of NGS data alongside histological subtype, grade, MSI/MMR status, menopausal status, co-mutation patterns, and TCGA-inspired molecular classification within a single-center, real-world cohort. Particularly, the increasing variant burden with increasing grade in endometrioid carcinomas and the *TP53*-dominant profile in serous carcinomas stand out as robust findings consistent with the literature and biologically meaningful. Furthermore, the recurrent alterations within the *BLM*, *CDC27*, *MLH3*, and *MSH3* axis provide novel observations that may warrant further investigation in prospective studies.

Nevertheless, several limitations of this study should be acknowledged. First, this was a retrospective, single-center study with a relatively limited sample size. In particular, the small number of premenopausal patients (*n* = 8) and the limited number of Grade 1 endometrioid tumors reduced the statistical power of subgroup analyses. Therefore, comparisons based on menopausal status and certain histological or grade subgroups should be considered exploratory, and negative findings should be interpreted with caution. Second, menopausal status was determined according to age rather than documented clinical menopausal information because these data were not consistently available in the retrospective cohort. Although this approach has been used in previous retrospective studies, some degree of patient misclassification cannot be excluded. Third, because of the relatively small sample size and the low frequency of several genetic alterations, multivariable analyses were not performed, as reliable model estimation could not be achieved. In addition, several nominally significant gene-level and co-mutation associations lost statistical significance after correction for multiple comparisons, indicating that these findings require validation in larger independent cohorts. Fourth, molecular subgroup classification was established using a targeted 71-gene NGS panel together with MSI-PCR and MMR immunohistochemistry rather than whole-exome or whole-genome sequencing. Consequently, genome-wide copy-number alterations and comprehensive mutational signatures could not be evaluated, and the molecular subgroup assignment should be regarded as a TCGA-inspired surrogate classification rather than a complete TCGA classification. Accordingly, the molecular heterogeneity described in this study reflected targeted panel-based profiling rather than comprehensive genomic characterization. Fifth, although variant interpretation was performed using ACMG criteria and QCI™ annotation, matched normal tissue or peripheral blood samples were not available for paired analysis. Therefore, definitive discrimination between somatic and germline variants could not be achieved for all detected variants. Sixth, the functional significance of recurrent alterations, particularly in BLM, CDC27, MLH3, and MSH3, was not experimentally validated. Moreover, these variants could not be confirmed by an independent molecular method such as Sanger sequencing or droplet digital PCR because of the limited availability of residual FFPE tissue and DNA. Finally, comprehensive clinicopathological outcome data, including FIGO stage, adjuvant treatment, recurrence, survival, treatment response, and long-term follow-up, were not available for all patients. Consequently, the prognostic significance of the identified TCGA-inspired molecular subgroups could not be evaluated. Future multicenter prospective studies integrating comprehensive clinical outcome data, functional validation, and broader genomic profiling will be essential to confirm and extend the present findings.

## 5. Conclusions

In this cohort, molecular alterations appeared to be more closely associated with histological subtype and tumor grade than with menopausal status. However, the limited number of premenopausal patients warrants cautious interpretation of menopause-related findings, which should be considered exploratory until validated in larger cohorts. After BH-FDR correction, only the association between *TP53* alterations and serous histology remained statistically significant, further supporting the distinct molecular profile of serous endometrial carcinoma. In contrast, the associations observed for *CTNNB1*, *KRAS*, and several co-mutation patterns did not retain statistical significance after multiple-testing correction, although their distribution was consistent with previously reported molecular characteristics of endometrioid tumors. Likewise, the recurrent alterations involving *BLM*, *CDC27*, *MLH3*, and *MSH3* represent intriguing molecular findings in this cohort; however, given that these associations did not remain significant after BH-FDR correction, they should be regarded as exploratory and require validation in larger, independent patient cohorts.

## Figures and Tables

**Table 1:** Clinicopathological and Molecular Characteristics of the Study Cohort (*n* = 81).

Variable	Total Cohort (*n* = 81)
Age (years), mean ± SD	63.3 ± 9.4
**Menopausal status, *n* (%)**	
Premenopausal	8 (9.9)
Postmenopausal	73 (90.1)
**Histological subtype, *n* (%)**	
Endometrioid carcinoma	57 (70.4)
Serous carcinoma	24 (29.6)
**Endometrioid tumor grade, *n* (%)**	
Grade 1	9 (15.8)
Grade 2	34 (59.6)
Grade 3	14 (24.6)
**Molecular characteristics**	
Total variant records	756
Variants per patient, mean ± SD	9.33 ± 5.73
Altered genes per patient, mean ± SD	7.02 ± 4.17
**MMR/MSI status, *n* (%)**	
*MLH1* loss	18 (22.2)
*PMS2* loss	17 (21.0)
*MSH2* loss	1 (1.2)
*MSH6* loss	2 (2.5)
MSS	60 (74.1)
MSI-L	0 (0.0)
MSI-H	18 (22.2)
Not available	3 (3.7)
MMRd/MSI-H	18 (22.2)
**TCGA-inspired molecular classification, *n* (%)**	
NSMP-proxy	27 (33.3)
*POLE*-mutated proxy	26 (32.1)
p53-abnormal proxy	19 (23.5)
MMRd/MSI-H	9 (11.1)

Loss of individual MMR proteins was not mutually exclusive, as some tumors showed concurrent loss of more than one MMR protein. Therefore, individual MMR protein-loss frequencies should not be summed to derive the number of unique MMR-deficient tumors.

**Table 2:** Molecular Alterations and MSI/MMR Findings According to Histological Subtype (BH-FDR Corrected).

Variable	Endometrioid Carcinoma (*n* = 57) *n* (%)	Serous Carcinoma (*n* = 24) *n* (%)	Raw *p*-Value	BH-FDR q Value
**Gene Alterations**				
*BLM*	44 (77.2)	21 (87.5)	0.371	0.795
*CDC27*	33 (57.9)	17 (70.8)	0.310	0.775
*MLH3*	30 (52.6)	7 (29.2)	0.074	0.277
*MSH3*	26 (45.6)	10 (41.7)	0.807	1.000
*PTEN*	25 (43.9)	8 (33.3)	0.463	0.846
*PIK3CA*	17 (29.8)	12 (50.0)	0.108	0.324
*POLE*	18 (31.6)	8 (33.3)	1.000	1.000
*TP53*	15 (26.3)	16 (66.7)	0.001	**0.015**
*CTNNB1*	11 (19.3)	0 (0.0)	0.028	0.140
*KRAS*	11 (19.3)	0 (0.0)	0.028	0.140
**MMR/MSI Findings**				
MLH1 loss	14 (24.6)	4 (16.7)	0.564	0.846
PMS2 loss	13 (22.8)	4 (16.7)	0.758	1.000
MSH2 loss	3 (5.3)	1 (4.2)	1.000	1.000
MSH6 loss	3 (5.3)	1 (4.2)	1.000	1.000
MMRd/MSI-H	14 (24.6)	4 (16.7)	0.564	0.846

*p* values were calculated using Pearson’s chi-square test or Fisher’s exact test, as appropriate, according to the expected cell counts. BH-FDR q values were calculated using the BH-FDR procedure. Bold value indicate statistical significance after BH-FDR correction (q < 0.05).

**Table 3:** Grade-Specific Molecular Alterations and MSI/MMR Findings in Endometrioid Carcinomas According to Tumor Grade (BH-FDR Corrected).

Variable	Grade 1 (*n* = 9) *n* (%)	Grade 2 (*n* = 34) *n* (%)	Grade 3 (*n* = 14) *n* (%)	*p*-Value	BH-FDR q Value
**Genomic Burden**					
Mean variants per case	4.44 ± 2.19	9.68 ± 5.37	11.42 ± 6.11	0.006	**0.030**
Mean altered genes per case	3.78 ± 1.39	7.41 ± 3.84	8.17 ± 4.72	0.021	0.0525
**Gene Alterations**					
*BLM*	3 (33.3)	29 (85.3)	10 (71.4)	0.004	**0.030**
*CDC27*	2 (22.2)	22 (64.7)	9 (64.3)	0.034	0.060
*MLH3*	1 (11.1)	22 (64.7)	7 (50.0)	0.016	0.0525
*MSH3*	1 (11.1)	17 (50.0)	8 (57.1)	0.036	0.060
*PTEN*	2 (22.2)	16 (47.1)	7 (50.0)	0.125	0.206
*PIK3CA*	1 (11.1)	11 (32.4)	5 (35.7)	0.154	0.220
*TP53*	2 (22.2)	9 (26.5)	4 (28.6)	0.901	0.901
**MMR/MSI Findings**					
MMRd/MSI-H	1 (11.1)	9 (26.5)	4 (28.6)	0.417	0.521

*p* values for continuous variables were calculated using one-way ANOVA. Categorical variables were compared using Pearson’s chi-square test, as appropriate according to the expected cell counts. BH-FDR q values were calculated using the BH-FDR procedure. Bold values indicate statistical significance after BH-FDR correction (q < 0.05).

**Table 4:** Co-Mutation Patterns According to Histological Subtype (BH-FDR Corrected).

Co-Mutation Pair	Overall Cohort *n* (%)	Endometrioid *n* (%)	Serous *n* (%)	Raw *p*-Value	BH-FDR q Value
*BLM–CDC27*	45 (55.6)	30 (52.6)	15 (62.5)	0.470	0.674
*BLM–MLH3*	34 (42.0)	27 (47.4)	7 (29.2)	0.147	0.323
*BLM–MSH3*	33 (40.7)	24 (42.1)	9 (37.5)	0.806	0.806
*CDC27–MSH3*	30 (37.0)	22 (38.6)	8 (33.3)	0.802	0.806
*CDC27–MLH3*	29 (35.8)	24 (42.1)	5 (20.8)	0.080	0.220
*BLM–PTEN*	28 (34.6)	21 (36.8)	7 (29.2)	0.613	0.749
*MLH3–MSH3*	27 (33.3)	22 (38.6)	5 (20.8)	0.196	0.359
*BLM–TP53*	26 (32.1)	13 (22.8)	13 (54.2)	0.009	**0.044**
*TP53–PIK3CA*	16 (19.8)	6 (10.5)	10 (41.7)	0.004	**0.044**
*CDC27–TP53*	21 (25.9)	10 (17.5)	11 (45.8)	0.012	**0.044**
*BLM–PIK3CA*	25 (30.9)	16 (28.1)	9 (37.5)	0.437	0.674

*p* values were calculated using Pearson’s chi-square test or Fisher’s exact test, as appropriate according to the expected cell counts. BH-FDR q values were calculated using the BH-FDR procedure. BH-FDR correction was applied across the 11 co-mutation comparisons included in this analysis. Bold values indicate statistical significance after BH-FDR correction (q < 0.05).

**Table 5:** TCGA-Inspired Molecular Classification According to Histological Subtype and Tumor Grade.

Molecular Subgroup	Total Cohort (*n* = 81) *n* (%)	Endometrioid (*n* = 57) *n* (%)	Serous (*n* = 24) *n* (%)	Grade 1 (*n* = 9) *n* (%)	Grade 2 (*n* = 34) *n* (%)	Grade 3 (*n* = 14) *n* (%)
NSMP-proxy	27 (33.3)	23 (40.4)	4 (16.7)	6 (66.7)	13 (38.2)	4 (28.6)
*POLE*-mutated	26 (32.1)	18 (31.6)	8 (33.3)	2 (22.2)	12 (35.3)	4 (28.6)
**p53**-abnormal proxy	19 (23.5)	9 (15.8)	10 (41.7)	0 (0.0)	5 (14.7)	4 (28.6)
MMRd/MSI-H	9 (11.1)	7 (12.3)	2 (8.3)	1 (11.1)	4 (11.8)	2 (14.3)
*p* value	—	0.012		0.084		
BH-FDR q-value		**0.012**		0.084		

Histological subgroup distributions were compared using Pearson’s χ^2^ test. BH-FDR q values were calculated using the BH-FDR procedure. Bold values indicate statistical significance after BH-FDR correction (q < 0.05).

## Data Availability

The data presented in this study are available on request from the corresponding author due to privacy and ethical restrictions. The data were obtained retrospectively from patient medical records, and individual patient information cannot be shared publicly to protect confidentiality.

## Supplementary Materials

The following supporting information can be downloaded at: https://www.mdpi.com/article/10.3390/biomedicines14091880/s1. Supplementary Figure S1. Complete oncoprint of all 81 endometrial cancer cases. Supplementary Table S1. Summary of unique pathogenic and likely pathogenic variants identified in the study cohort by targeted NGS. Supplementary Table S2. Detailed Characteristics of POLE Variants Identified in the Study Cohort. Supplementary Table S3. Pathogenic and Likely Pathogenic Variants Identified in POLE, TP53, BLM, CDC27, MLH3, and MSH3 Genes Including Variant Allele Frequency (VAF), Sequencing Coverage, and POLE Molecular Assignment. Supplementary Table S4. Overlap of Molecular Classifiers Before Hierarchical TCGA-Inspired Classification. Supplementary Table S5. Variant Burden According to TCGA-Inspired Molecular Classification Subgroups.

## Abbreviations

The following abbreviations are used in this manuscript

ACMG	American College of Medical Genetics and Genomics
FFPE	Formalin-fixed paraffin-embedded
FIGO	International Federation of Obstetrics and Gynecology
MMR	Mismatch repair
MSI	Microsatellite instability
MSS	Microsatellite stable
NGS	Next-generation sequencing
NSMP	No specific molecular profile
TCGA	The Cancer Genome Atlas
QCI	Qiagen Clinical Insight Interpret
VAF	Variant allele frequency
VUS	Variant of uncertain significance
