# Supplementary material for: Integrated Molecular Profiling of Endometrial Cancer: NGS Findings, MSI/MMR Status, Histopathological Features, and TCGA-Inspired Molecular Classification
Source: Biomedicines. 2026 Aug 23;14(9):1880. doi: 10.3390/biomedicines14091880 (PMC13604228; doi:10.3390/biomedicines14091880)
Supplement: Supplementary file 1 [file biomedicines-14-01880-s001.zip › biomedicines-4439877-supplementary.pdf]

Supplementary Figure S1. Complete oncoprint of all 81 endometrial cancer cases. Columns represent individual tumor samples (Case ID1–Case ID81), and rows represent genes harboring pathogenic or likely pathogenic variants identified by targeted next-generation sequencing. Colored boxes indicate the mutation type detected in each tumor, including missense/single nucleotide variants (SNVs), frameshift variants, nonsense variants, splice-site variants, and in-frame insertions/deletions. Cases with multiple pathogenic variants in the same gene are displayed as multiple-hit events. The upper bar plot shows the total number of pathogenic or likely pathogenic variants detected in each tumor. The annotation tracks indicate histological subtype, tumor grade, menopausal status, and MSI/MMR status for each case. The bar plot on the right shows the frequency of alterations for each gene across the entire cohort (n = 81). Genes are ordered according to the frequency of pathogenic or likely pathogenic alterations

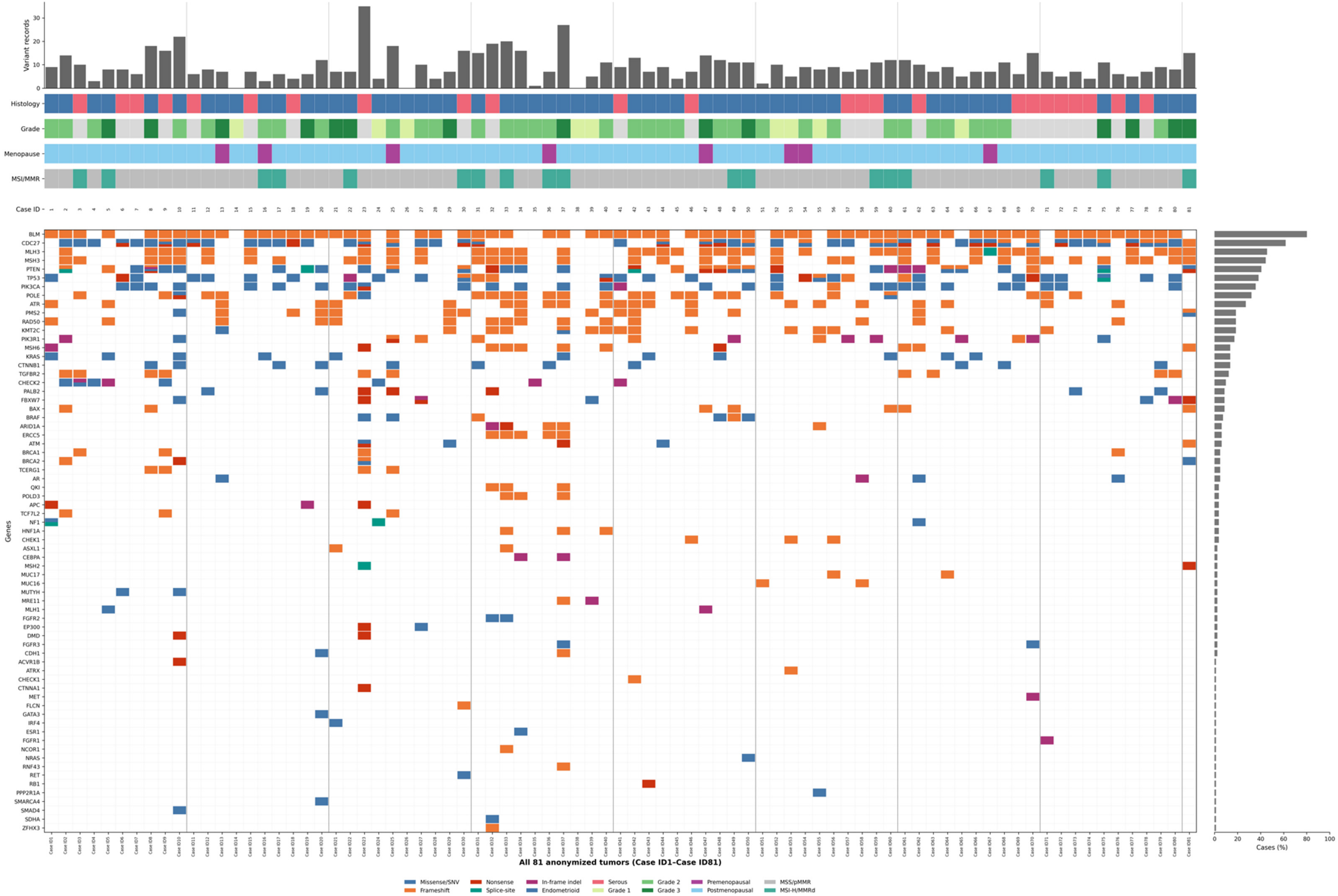
Variant records
Histology
Grade
Menopause
MSI/MMR
Case ID
BLM
CDC27
MLH3
MSH3
PTEN
TP53
PIK3CA
POLE
ATR
PMS2
RAD50
KMT2C
PIK3R1
MSH6
KRAS
CTNNB1
TGFBR2
CHECK2
PALB2
FBXW7
BAX
BRAF
ARID1A
ERCC5
ATM
BRCA1
BRCA2
TCERG1
AR
QKI
POLD3
APC
TCF7L2
NF1
HNF1A
CHEK1
ASXL1
CEBPA
MSH2
MUC17
MUC16
MUTYH
MRE11
MLH1
FGFR2
EP300
DMD
FGFR3
CDH1
ACVR1B
ATRX
CHECK1
CTNNA1
MET
FLCN
GATA3
IRF4
ESR1
FGFR1
NCOR1
NRAS
RNF43
RET
RB1
PPP2R1A
SMARCA4
SMAD4
SDHA
ZFHX3
Genes
All 81 anonymized tumors (Case ID1–Case ID81)
Cases (%)
Missense/SNV
Frameshift
Nonsense
Splice-site
In-frame indel
Endometrioid
Serous
Grade 1
Grade 2
Grade 3
Premenopausal
Postmenopausal
MSS/pMMR
MSI-H/MMRd

Supplementary Table S1. Summary of unique pathogenic and likely pathogenic variants identified in the study cohort by targeted NGS.

Gene	Exon	HGVS c. Nomenclature	HGVS p. Nomenclature	Variant Type	ACMG Classification	Frequency, n (%)
ACVR1B	6	c.1000C>T	p.R334*	Nonsense (stop-gain)	Likely pathogenic	1 (1.2%)
ACVR1B	7	c.1162G>T	p.E388*	Nonsense (stop-gain)	Likely pathogenic	1 (1.2%)
APC	10	c.1170_1172delCAT	p.I391del	In-frame deletion	Likely pathogenic	1 (1.2%)
APC	16	c.4348C>T	p.R1450*	Nonsense (stop-gain)	Pathogenic	1 (1.2%)
APC	16	c.575C>T	p.R1920*	Nonsense (stop-gain)	Likely pathogenic	1 (1.2%)
APC	16	c.6676C>T	p.R2226*	Nonsense (stop-gain)	Likely pathogenic	1 (1.2%)
AR	1	c.170T>A	p.L57Q	Missense	Pathogenic	2 (2.5%)
AR	1	c.173A>T	p.Q58L	Missense	Likely pathogenic	1 (1.2%)
AR	1	c.271_273del	p.Q91del	In-frame deletion	Likely pathogenic	1 (1.2%)
ARID1A	1	c.275_291del	p.G92AFS*13	Frameshift deletion	Likely pathogenic	1 (1.2%)
ARID1A	1	c.60_62delGCC	p.P21del	In-frame deletion	Likely pathogenic	1 (1.2%)
ARID1A	20	c.5548del	p.D1850Tfs*33	Frameshift deletion	Pathogenic	1 (1.2%)
ARID1A	20	c.5548dup	p.D1850Gfs*4	Frameshift insertion	Pathogenic	1 (1.2%)
ARID1A	20	c.6598G>T	p.E2200*	Nonsense (stop-gain)	Likely pathogenic	1 (1.2%)
ASXL1	13	c.1934dup	p.G646fs*12	Frameshift insertion	Pathogenic	2 (2.5%)
ATM	8	c.1009C>T	p.R337C	Missense	Likely pathogenic	1 (1.2%)
ATM	14	c.2143_2144del	p.L715Cfs*22	Frameshift deletion	Likely pathogenic	1 (1.2%)
ATM	16	c.2413C>T	p.R805*	Nonsense (stop-gain)	Pathogenic	1 (1.2%)
ATM	24	c.3576G>A	p.K1192K	Synonymous	Pathogenic	2 (2.5%)
ATM	29	c.4273C>T	p.Q1425*	Nonsense (stop-gain)	Likely pathogenic	1 (1.2%)
ATM	63	c.9023G>A	p.R3008H	Missense	Pathogenic	1 (1.2%)
ATR	7/10	c.2320del	p.I774Yfs*5	Frameshift deletion	Pathogenic	18 (22.2%)
ATR	10	c.1544del	p.N515Mfs*16	Frameshift deletion	Pathogenic	1 (1.2%)
ATR	10	c.2320dup	p.I774Nfs*3	Frameshift insertion	Pathogenic / Likely pathogenic	8 (9.9%)
ATRX	9	c.1457dup	p.N486Kfs*2	Frameshift insertion	Likely pathogenic	2 (2.5%)
BAX	3	c.121del	p.E41Rfs*19	Frameshift deletion	Pathogenic	6 (7.4%)
BAX	3	c.121dup	p.E41Gfs*33	Frameshift insertion	Pathogenic	2 (2.5%)
BLM	3	c.520delC	p.Q174fs*5	Frameshift deletion	Likely pathogenic	1 (1.2%)
BLM	7	C.1544del	p.N515Mfs*16	Frameshift deletion	Pathogenic	65 (80.2%)
BRAF	10	c.1208del	p.P403fs*8	Frameshift deletion	Likely pathogenic	2 (2.5%)
BRAF	15	c.1787G>A	p.G596D	Missense	Pathogenic	1 (1.2%)
BRAF	17	c.2000T>G	p.F667C	Missense	Likely pathogenic	1 (1.2%)
BRAF	17	c.2045G>A	p.R682Q	Missense	Likely pathogenic	1 (1.2%)

BRAF	18	c.2141T>A	p.I714N	Missense	Likely pathogenic	1 (1.2%)
BRCA1	2	c.66dup	p.E23Rfs*18	Frameshift insertion	Pathogenic	1 (1.2%)
BRCA1	10	c.1961delA	p.K654fs*47	Frameshift deletion	Pathogenic	3 (3.7%)
BRCA2	11	c.2596G>T	p.E866*	Nonsense (stop-gain)	Likely pathogenic	2 (2.5%)
BRCA2	11	c.5073delA	p.K1691fs*15	Frameshift deletion	Pathogenic	1 (1.2%)
BRCA2	11	c.6058G>A	p.E2020K	Missense	Likely pathogenic	1 (1.2%)
BRCA2	11	c.6562A>G	p.K2188E	Missense	Likely pathogenic	1 (1.2%)
BRCA2	18	c.8009C>T	p.S2670L	Missense	Pathogenic	1 (1.2%)
BRCA2	23	c.9097delA	p.T3033fs*29	Frameshift deletion	Pathogenic	2 (2.5%)
CDC27	1	c.17A>G	p.E6G	Missense	Likely pathogenic	1 (1.2%)
CDC27	1	c.4A>G	p.T2A	Missense	Likely pathogenic	1 (1.2%)
CDC27	2	c.60T>C	p.A20A	Synonymous	Likely pathogenic	1 (1.2%)
CDC27	3	c.128delT	p.L43fs*19	Frameshift deletion	Likely pathogenic	2 (2.5%)
CDC27	3	c.204A>C	p.T68T	Synonymous	Likely pathogenic	29 (35.8%)
CDC27	3/7	c.218A>G	p.Y73C	Missense	Pathogenic	35 (43.2%)
CDC27	3/7	c.228A>G	p.A76A	Synonymous	Likely pathogenic	27 (33.3%)
CDC27	3	c.2726_2727del	p.E909Afs*17	Frameshift deletion	Pathogenic	1 (1.2%)
CDC27	6	c.524A>G	p.N175S	Missense	Likely pathogenic	1 (1.2%)
CDC27	6	c.524G>A	p.N175S	Missense	Likely pathogenic	1 (1.2%)
CDC27	7/27	c.761T>G	p.L254*	Nonsense (stop-gain)	Pathogenic	16 (19.8%)
CDC27	7	c.778A>C	p.N260H	Missense	Pathogenic	16 (19.8%)
CDC27	12	c.1512_1513insC	p.G505fs*6	Frameshift insertion	Likely pathogenic	1 (1.2%)
CDC27	12/13	c.1513dup	p.I505Nfs*7	Frameshift insertion	Likely pathogenic	4 (4.9%)
CDC27	12	c.1515_1516insC	p.G505fs*6	Frameshift insertion	Likely pathogenic	8 (9.9%)
CDC27	13	c.1677A>C	p.L559F	Missense	Likely pathogenic	3 (3.7%)
CDC27	14	c.1732A>G	p.S578G	Missense	Likely pathogenic	1 (1.2%)
CDC27	14	c.1777G>A	p.A593T	Missense	Likely pathogenic	1 (1.2%)
CDC27	14	c.1905T>G	p.N635K	Missense	Likely pathogenic	1 (1.2%)
CDC27	14	c.1908T>G	p.N636K	Missense	Likely pathogenic	4 (4.9%)
CDH1	3	c.377del	p.P126Rfs*89	Frameshift deletion	Likely pathogenic	1 (1.2%)
CDH1		Exon 1-2 deletion		Other	Pathogenic	1 (1.2%)
CEBPA	1	c.564_566del	p.P189del	In-frame deletion	Pathogenic	2 (2.5%)
CHEK1	7	c.676del	p.T226Hfs*14	Frameshift deletion	Pathogenic / Likely pathogenic	4 (4.9%)
CHEK2	11	c.1116_1117delins TG	p.K373E	In-frame deletion-insertion	Pathogenic	2 (2.5%)
CHEK2	13	c.1148delA	p.E493K	In-frame deletion	Likely pathogenic	1 (1.2%)
CHEK2	13	c.1477G>A	p.E493K	Missense	Likely pathogenic	3 (3.7%)
CHEK2	14	c.1552_1524delAA G	p.K508del	In-frame deletion	Likely pathogenic	1 (1.2%)
CHEK2	14	c.15556C>T	p.T519M	Missense	Pathogenic	4 (4.9%)
CTNNA1	8	c.1075G>T	p.E359*	Nonsense (stop-gain)	Likely pathogenic	1 (1.2%)
CTNNB1	3	c.100G>A	p.G34R	Missense	Pathogenic	1 (1.2%)
CTNNB1	3	c.101G>A	p.G34E	Missense	Pathogenic	1 (1.2%)
CTNNB1	3	c.110C>G	p.S37C	Missense	Pathogenic	2 (2.5%)
CTNNB1	3	c.110C>T	p.S375	Other	Pathogenic	2 (2.5%)

CTNNB1	3	c.122C>T	p.T41I	Missense	Pathogenic	2 (2.5%)
CTNNB1	3	c.947C>T	p.D32Y	Missense	Pathogenic	1 (1.2%)
CTNNB1	3	c.94G>A	p.D32N	Missense	Pathogenic	2 (2.5%)
CTNNB1	3	c.94G>T	p.D32Y	Missense	Pathogenic	1 (1.2%)
CTNNB1	3	c.98C>G	p.S33C	Missense	Pathogenic	1 (1.2%)
DMD	6	c.409C>T	p.R137*	Nonsense (stop-gain)	Likely pathogenic	2 (2.5%)
DMD	25	c.3397G>T	p.E1133*	Nonsense (stop-gain)	Likely pathogenic	1 (1.2%)
DMD	33	c.4618G>T	p.E1540*	Nonsense (stop-gain)	Likely pathogenic	1 (1.2%)
DMD	42	c.6013G>T	p.E2005*	Nonsense (stop-gain)	Likely pathogenic	1 (1.2%)
EP300	2	c.256C>T	p.R86*	Nonsense (stop-gain)	Likely pathogenic	1 (1.2%)
EP300	2	c.631G>A	p.G211S	Missense	Pathogenic	1 (1.2%)
ERCC5	13	c.2751del	p.K917fs*65	Frameshift deletion	Pathogenic	5 (6.2%)
ESR1	8	c.1607T>C	p.L536P	Missense	Pathogenic	1 (1.2%)
FBXW7	4	c.349_351delGAG	p.E117del	In-frame deletion	Likely pathogenic	1 (1.2%)
FBXW7	5	c.585-7_585-5del		In-frame deletion	Likely pathogenic	1 (1.2%)
FBXW7	6	c.670C>T	p.R224*	Nonsense (stop-gain)	Pathogenic	1 (1.2%)
FBXW7	11	c.1393C>T	p.R465C	Missense	Pathogenic	2 (2.5%)
FBXW7	11	c.1394G>A	p.R465H	Missense	Pathogenic	1 (1.2%)
FBXW7	12	c.1436G>A	p.R479Q	Missense	Pathogenic	1 (1.2%)
FBXW7	12/14	c.1972C>T	p.R658*	Nonsense (stop-gain)	Pathogenic	2 (2.5%)
FGFR1	4	c.396_398del	p.D133del	In-frame deletion	Pathogenic	1 (1.2%)
FGFR2	2	c.755C>G	p.S252W	Missense	Pathogenic	1 (1.2%)
FGFR2	9	c.1144T>C	p.C382R	Missense	Pathogenic	2 (2.5%)
FGFR3	14	c.1924G>A	p.V642M	Missense	Likely pathogenic	1 (1.2%)
FGFR3	17	c.2261T>G	p.V754G	Missense	Likely pathogenic	1 (1.2%)
FLCN	11	c.1285dupC	p.H429fs*27	Frameshift insertion	Pathogenic	1 (1.2%)
GATA3		Exon 2-3 deletion		Other	Pathogenic	1 (1.2%)
HNF1A	4	c.864del	p.P291Qfs*51	Frameshift deletion	Pathogenic	1 (1.2%)
HNF1A	4	c.872del	p.P291Qfs*51	Frameshift deletion	Pathogenic	2 (2.5%)
HNF1A	4	c.872dup	p.G292Rfs*25	Frameshift insertion	Pathogenic	1 (1.2%)
IRF4	3	c.368A>G	p.K123R	Missense	Pathogenic	1 (1.2%)
KMT2C	7	c.1544delA	p.N515fs*16	Frameshift deletion	Pathogenic	1 (1.2%)
KMT2C	7	c.925C>T	p.P309S	Missense	Pathogenic	1 (1.2%)
KMT2C	18	c.2968A>G	p.S990G	Missense	Pathogenic	1 (1.2%)
KMT2C	38	c.8390del	p.K2797Rfs*26	Frameshift deletion	Pathogenic	14 (17.3%)
KRAS	2	c.34G>T	p.G12C	Missense	Pathogenic	1 (1.2%)
KRAS	2	c.35G>A	p.G12D	Missense	Pathogenic	1 (1.2%)
KRAS	2	c.35G>C	p.G12A	Missense	Pathogenic	2 (2.5%)
KRAS	2	c.35G>T	p.G12V	Missense	Pathogenic	4 (4.9%)
KRAS	2	c.57G>A	p.L19F	Missense	Pathogenic	1 (1.2%)
KRAS	4	c.436G>A	p.A146T	Missense	Pathogenic	2 (2.5%)
MET	13	c.2888-55_288-4del		In-frame deletion	Likely pathogenic	1 (1.2%)
MLH1	1	c.62_70del	p.A21_E23del	In-frame deletion	Likely pathogenic	1 (1.2%)
MLH1	2	c.199G>A	p.G67R	Missense	Pathogenic	1 (1.2%)

MLH3	2	c.1043dupT	p.L348fs*13	Frameshift insertion	Likely pathogenic	1 (1.2%)
MLH3	2	c.1755del	p.E586Nfs*24	Frameshift deletion	Pathogenic / Likely pathogenic	36 (44.4%)
MLH3	2	c.2021del	p.N674Ifs*6	Frameshift deletion	Pathogenic / Likely pathogenic	4 (4.9%)
MLH3	6	c.3571-1G>T		Splice-site	Likely pathogenic	1 (1.2%)
MRE11	4	c.315-4del		In-frame deletion	Pathogenic	1 (1.2%)
MRE11	14	c.1532del	p.N511Ifs*13	Frameshift deletion	Pathogenic	1 (1.2%)
MSH2	15	c.2459-1G>T	p.-	Splice-site	Likely pathogenic	1 (1.2%)
MSH2	15	c.2470C>T	p.Q824*	Nonsense (stop-gain)	Pathogenic	1 (1.2%)
MSH3	3	c.408del	p.K136Nfs*2	Frameshift deletion	Likely pathogenic	1 (1.2%)
MSH3	7	c.1148del	p.K383Rfs*32	Frameshift deletion	Pathogenic	36 (44.4%)
MSH6	4	c.1921G>T	p.E641*	Nonsense (stop-gain)	Likely pathogenic	1 (1.2%)
MSH6	4	c.642C>A	p.Y214*	Nonsense (stop-gain)	Pathogenic	1 (1.2%)
MSH6	5	c.3261del	p.F1088Sfs*2	Frameshift deletion	Pathogenic	6 (7.4%)
MSH6	5	c.3261dup	p.F1088Lfs*5	Frameshift insertion	Pathogenic	2 (2.5%)
MSH6	5	c.326delC	p.F1088fs*2	Frameshift deletion	Pathogenic	1 (1.2%)
MSH6	5	c.3312del	p.F1104Lfs*11	Frameshift deletion	Pathogenic	1 (1.2%)
MSH6	8	c.3668_3670delAT Gp.D1223del	p.	In-frame deletion	Likely pathogenic	1 (1.2%)
MUC16	8	c.32863_33019del	p.E10955Pfs*22	Frameshift deletion	Likely pathogenic	1 (1.2%)
MUC16	8	c.32980_33019del	p.F10994Pfs*22	Frameshift deletion	Likely pathogenic	1 (1.2%)
MUC16	8	c.33027_33031del	p.G11010Rfs*12	Frameshift deletion	Likely pathogenic	1 (1.2%)
MUC16	8	c.33035_33141del	p.E11012Afs*5	Frameshift deletion	Likely pathogenic	1 (1.2%)
MUC17	3	c.10113_10134del	p.T3372Yfs*23	Frameshift deletion	Likely pathogenic	1 (1.2%)
MUC17	3	c.3699_3750del	p.S1233Rfs*31	Frameshift deletion	Likely pathogenic	1 (1.2%)
MUTYH	9	c.650G>A	p.R217H	Missense	Pathogenic	1 (1.2%)
MUTYH	12	c.1069G>C	p.A357P	Missense	Likely pathogenic	1 (1.2%)
NCOR1	15	c.1584dup	p.E529Rfs*26	Frameshift insertion	Pathogenic	1 (1.2%)
NF1	13	c.1400C>T	p.T467I	Missense	Pathogenic	1 (1.2%)
NF1	16	c.1759A>T	p.S587C	Missense	Likely pathogenic	1 (1.2%)
NF1	19	c.2325+3A>G		Splice-site	Pathogenic	2 (2.5%)
NRAS	2	c.35G>A	p.G12D	Missense	Pathogenic	1 (1.2%)
PALB2	4	c.886delA	p.M296*	In-frame deletion	Pathogenic	2 (2.5%)
PALB2	9	c.2896A>G	p.I966V	Missense	Likely pathogenic	1 (1.2%)
PALB2	12	c.3203G>A	p.G1068E	Missense	Likely pathogenic	1 (1.2%)
PALB2	12	c.3256C>T	p.R1086*	Nonsense (stop-gain)	Pathogenic	1 (1.2%)
PALB2	12	c.3306C>G	p.S1102R	Missense	Likely pathogenic	1 (1.2%)
PALB2		Exon 1 deletion		Other	Pathogenic	1 (1.2%)
PIK3CA	2	c.241G>A	p.E81K	Missense	Pathogenic	1 (1.2%)
PIK3CA	2	c.263G>A	p.R1086*	Nonsense (stop-gain)	Pathogenic	1 (1.2%)
PIK3CA	2	c.263G>A	p.R88Q	Missense	Pathogenic	1 (1.2%)
PIK3CA	2	c.277C>T	p.R93W	Missense	Pathogenic	1 (1.2%)
PIK3CA	2	c.317G>T	p.G106V	Missense	Pathogenic	1 (1.2%)
PIK3CA	2	c.323G>A	p.R108H	Missense	Pathogenic	1 (1.2%)

PIK3CA	3	c.353G>A	p.G118D	Missense	Pathogenic	1 (1.2%)
PIK3CA	5	c.1032_1037dup	p.V346_N347dup	In-frame duplication	Likely pathogenic	1 (1.2%)
PIK3CA	5	c.1035T>A	p.N345K	Missense	Pathogenic	1 (1.2%)
PIK3CA	6	c.1093G>A	p.E365K	Missense	Pathogenic	1 (1.2%)
PIK3CA	8	c.1258T>C	p.C420R	Missense	Pathogenic	1 (1.2%)
PIK3CA	10	c.1624G>A	p.E542K	Missense	Pathogenic	2 (2.5%)
PIK3CA	10	c.1625A>T	p.E542V	Missense	Pathogenic	1 (1.2%)
PIK3CA	10	c.1633G>A	p.E545K	Missense	Pathogenic	4 (4.9%)
PIK3CA	10	c.1635G>T	p.E545D	Missense	Pathogenic	1 (1.2%)
PIK3CA	10	c.1637A>C	p.Q546P	Missense	Pathogenic	1 (1.2%)
PIK3CA	10	c.1653G>C	p.E545D	Missense	Pathogenic	1 (1.2%)
PIK3CA	14	c.2176G>A	p.E726K	Missense	Pathogenic	3 (3.7%)
PIK3CA	19	c.2740G>A	p.G914R	Missense	Pathogenic	1 (1.2%)
PIK3CA	21	c.3019G>C	p.G1007R	Missense	Pathogenic	1 (1.2%)
PIK3CA	21	c.3062A>C	p.Y1021S	Missense	Likely pathogenic	1 (1.2%)
PIK3CA	21	c.3140A>C	p.H1047R	Missense	Pathogenic	1 (1.2%)
PIK3CA	21	c.3140A>G	p.H1047R	Missense	Pathogenic	5 (6.2%)
PIK3CA	21	c.3140A>G	p.N515Mfs*16H1047R	Frameshift	Pathogenic	1 (1.2%)
PIK3R1	2	c.244delA	p.I82fs*32	Frameshift deletion	Likely pathogenic	1 (1.2%)
PIK3R1	10	c.1187dupT	p.L396fs?8	Frameshift insertion	Likely pathogenic	1 (1.2%)
PIK3R1	10	c.1193dup	p.S399Qfs*5	Frameshift insertion	Likely pathogenic	1 (1.2%)
PIK3R1	10	c.1196_1213del	p.S399_L404del	In-frame deletion	Likely pathogenic	1 (1.2%)
PIK3R1	10	c.1239_1299+23del		In-frame deletion	Likely pathogenic	1 (1.2%)
PIK3R1	11	c.1327_1343del	p.E443Ifs*13	Frameshift deletion	Likely pathogenic	1 (1.2%)
PIK3R1	11	c.1348_1357del	p.H450Tfs*27	Frameshift deletion	Likely pathogenic	1 (1.2%)
PIK3R1	11	c.1350_1370del	p.H450_F456del	In-frame deletion	Likely pathogenic	1 (1.2%)
PIK3R1	13	c.1690A>G	p.N564D	Missense	Pathogenic	2 (2.5%)
PIK3R1	13	c.1707_1709dup	p.L570dup	In-frame duplication	Likely pathogenic	1 (1.2%)
PIK3R1	13	c.1712_1720dupTCCAG	p.I571_L573dup	In-frame duplication	Likely pathogenic	1 (1.2%)
PIK3R1	13	c.1723_1724delAA	p.K575fs*26	Frameshift deletion	Likely pathogenic	1 (1.2%)
PIK3R1	13	c.1746-10_1767del		In-frame deletion	Likely pathogenic	1 (1.2%)
PIK3R1	15	c.1915C>T	p.R639*	Nonsense (stop-gain)	Likely pathogenic	1 (1.2%)
PIK3R1	16	c.2008_2012dup	p.I672Vfs*3	Frameshift insertion	Likely pathogenic	1 (1.2%)
PIK3R1	16	c.2019_2022del	p.N673Kfs*18	Frameshift deletion	Likely pathogenic	1 (1.2%)
PMS2	2	c.113C>T	p.A38V	Missense	Likely pathogenic	1 (1.2%)
PMS2	5	c.506G>A	p.R169H	Missense	Likely pathogenic	1 (1.2%)
PMS2	11	c.1239del	p.D414Tfs*34	Frameshift deletion	Pathogenic / Likely pathogenic	14 (17.3%)
POLD3	8	c.898del	p.R300Gfs*5	Frameshift deletion	Pathogenic	3 (3.7%)
POLE	4	c.309T>G	p.Y103*	Nonsense (stop-gain)	Likely pathogenic	1 (1.2%)
POLE	8	c.968dup	p.N323Kfs*2	Frameshift insertion	Pathogenic	1 (1.2%)
POLE	11	c.1082C>T	p.T361I	Missense	Likely pathogenic	1 (1.2%)
POLE	13	c.1231G>T	p.V411L	Missense	Pathogenic	1 (1.2%)

POLE	14	c.1394C>T	p.A465V	Missense	Likely pathogenic	1 (1.2%)
POLE	34	c.4337_4338delTG	p.V1446fs*3	Frameshift deletion	Pathogenic	21 (25.2%)
PPP2R1A	5	c.536C>G	p.P179R	Missense	Pathogenic	1 (1.2%)
PTEN	1	c.17_18del	p.K6Rfs*4	Frameshift deletion	Pathogenic	1 (1.2%)
PTEN	1	c.46T>G	p.Y16D	Missense	Likely pathogenic	1 (1.2%)
PTEN	1	c.48t>A	p.Y16*	Nonsense (stop-gain)	Pathogenic	1 (1.2%)
PTEN	2	c.106G>A	p.G36R	Missense	Pathogenic	1 (1.2%)
PTEN	2	c.113C>G	p.P38R	Missense	Pathogenic	1 (1.2%)
PTEN	2	c.120_123del	p.E40Dfs*13	Frameshift deletion	Likely pathogenic	1 (1.2%)
PTEN	3	c.193T>G	p.Y65D	Missense	Pathogenic	1 (1.2%)
PTEN	4	c.210-2A>G		Splice-site	Likely pathogenic	1 (1.2%)
PTEN	4	c.210-2A>T		Splice-site	Likely pathogenic	1 (1.2%)
PTEN	4	c.226_228delTAT	p.Y76del	In-frame deletion	Pathogenic	1 (1.2%)
PTEN	5	c.323T>C	p.L108P	Missense	Pathogenic	1 (1.2%)
PTEN	5	c.376G>A	p.A126T	Missense	Pathogenic	1 (1.2%)
PTEN	5	c.377C>A	p.A126D	Missense	Pathogenic	1 (1.2%)
PTEN	5	c.380G>T	p.G127V	Missense	Pathogenic	1 (1.2%)
PTEN	5	c.388C>G	p.R130G	Missense	Pathogenic	4 (4.9%)
PTEN	5	c.388C>T	p.R130*	Nonsense (stop-gain)	Pathogenic	2 (2.5%)
PTEN	5	c.389G>A	p.R130Q	Missense	Pathogenic	3 (3.7%)
PTEN	5	c.390_392del	p.T131del	In-frame deletion	Pathogenic	1 (1.2%)
PTEN	5	c.397G>A	p.V133I	Missense	Likely pathogenic	4 (4.9%)
PTEN	5	c.407G>A	p.C136Y	Missense	Pathogenic	5 (6.2%)
PTEN	5	c.432del	p.K144Nfs*3	Frameshift deletion	Likely pathogenic	1 (1.2%)
PTEN	6	c.504_505insA	p.P169fs*11	Frameshift insertion	Likely pathogenic	1 (1.2%)
PTEN	6	c.511C>T	p.Q171*	Nonsense (stop-gain)	Pathogenic	1 (1.2%)
PTEN	6	c.518G>A	p.R173H	Missense	Pathogenic	1 (1.2%)
PTEN	6	c.562_570del	p.Y188_P190del	In-frame deletion	Likely pathogenic	1 (1.2%)
PTEN	6	c.600delT	p.F200fs*21	Frameshift deletion	Pathogenic	1 (1.2%)
PTEN	7	c.645dupT	p.V216fs*27	Frameshift insertion	Pathogenic	1 (1.2%)
PTEN	7	c.672dup	p.Y225Ifs*18	Frameshift insertion	Pathogenic	1 (1.2%)
PTEN	7	c.724G>T	p.E242*	Nonsense (stop-gain)	Pathogenic	1 (1.2%)
PTEN	7	c.728_729insA	p.F243Lfs*10	Frameshift insertion	Likely pathogenic	1 (1.2%)
PTEN	7	c.740_741dupTA	p.P248fs*9	Frameshift insertion	Likely pathogenic	1 (1.2%)
PTEN	7	c.752G>T	p.G521V	Missense	Pathogenic	1 (1.2%)
PTEN	7	c.771C>A	p.F257L	Missense	Likely pathogenic	1 (1.2%)
PTEN	7	c.800del	p.K267Rfs*9	Frameshift deletion	Pathogenic	3 (3.7%)
PTEN	8	c.802-1G>A		Splice-site	Likely pathogenic	1 (1.2%)
PTEN	8	c.802-2_802-1delinsGA		In-frame deletion-insertion	Likely pathogenic	1 (1.2%)
PTEN	8	c.813dup	p.H272Sfs*26	Frameshift insertion	Likely pathogenic	1 (1.2%)
PTEN	8	c.867del	p.V290*	In-frame deletion	Pathogenic	2 (2.5%)

PTEN	8	c.878del	p.G293Efs*14	Frameshift deletion	Likely pathogenic	1 (1.2%)
PTEN	8	c.955_958delACTT	p.T319*	In-frame deletion	Pathogenic	2 (2.5%)
PTEN	9	c.1027-2A>G		Splice-site	Pathogenic	1 (1.2%)
PTEN	9	c.1114delAinsCAT	p.N372fs*	Frameshift insertion	Pathogenic	1 (1.2%)
PTEN		c.210_1G>A		Other	Pathogenic	1 (1.2%)
QKI	3	c.401del	p.K134fs*14	Frameshift deletion	Pathogenic	3 (3.7%)
RAD50	13	c.2165del	p.K722fs*14	Frameshift deletion	Pathogenic	13 (16.0%)
RAD50	13	c.2165dup	p.E723Gfs*5	Frameshift insertion	Pathogenic	1 (1.2%)
RAD50	13	c.2320del	p.K722Rfs*14	Frameshift deletion	Pathogenic	2 (2.5%)
RAD50	17	c.2801del	p.N934Ifs*6	Frameshift deletion	Pathogenic / Likely pathogenic	5 (6.2%)
RB1	17	c.1654C>T	p.R552*	Nonsense (stop-gain)	Pathogenic	1 (1.2%)
RET	11	c.1907C>T	p.T636M	Missense	Likely pathogenic	1 (1.2%)
RNF43	9	c.1976del	p.G659Vfs*41	Frameshift deletion	Pathogenic	1 (1.2%)
SDHA	10	c.1367C>T	p.S456L	Missense	Pathogenic	1 (1.2%)
SMAD4	10	c.1216G>A	p.A406T	Missense	Pathogenic	1 (1.2%)
SMARCA4		Exon 2-6 deletion		Other	Pathogenic	1 (1.2%)
TCERG1	18	c.2807delA	p.K936fs*17	Frameshift deletion	Likely pathogenic	4 (4.9%)
TCF7L2	14	c.1403delA	p.K468fs*23	Frameshift deletion	Pathogenic	3 (3.7%)
TGFBR2	3/13	c.383del	p.K128fs*35	Frameshift deletion	Pathogenic	10 (12.3%)
TP53	4	c.216del	p.V73Wfs*50	Frameshift deletion	Pathogenic	1 (1.2%)
TP53	4	c.318C>G	p.S106R	Missense	Pathogenic	1 (1.2%)
TP53	4	c.322G>T	p.G108C	Missense	Likely pathogenic	1 (1.2%)
TP53	4	c.375+1G>A		Splice-site	Pathogenic	1 (1.2%)
TP53	5	c.395A>G	p.K132R	Missense	Pathogenic	1 (1.2%)
TP53	5	c.422G>A	p.C141Y	Missense	Pathogenic	1 (1.2%)
TP53	5	c.455delC	p.P152fs*18	Frameshift deletion	Pathogenic	1 (1.2%)
TP53	5	c.516del	p.V173*	In-frame deletion	Pathogenic	1 (1.2%)
TP53	5	c.524G>A	p.R175H	Missense	Pathogenic	2 (2.5%)
TP53	5	c.532delC	p.H178fs*69	Frameshift deletion	Pathogenic	1 (1.2%)
TP53	5	c.535del	p.H179Mfs*68	Frameshift deletion	Pathogenic	1 (1.2%)
TP53	5	c.548C>G	p.S183*	Nonsense (stop-gain)	Pathogenic	1 (1.2%)
TP53	5	p.423C>G	p.C141W	Missense	Pathogenic	1 (1.2%)
TP53	6	c.578A>G	p.H193R	Missense	Pathogenic	3 (3.7%)
TP53	6	c.637C>T	p.R213*	Nonsense (stop-gain)	Pathogenic	2 (2.5%)
TP53	6	c.662_665dupAGCC	p.p223fs*3	Frameshift insertion	Pathogenic	1 (1.2%)
TP53	7	c.683_686del	p.D228Vfs*18	Frameshift deletion	Pathogenic	1 (1.2%)
TP53	7	c.729_730insAGC	p.M243_G244insS	In-frame insertion	Likely pathogenic	1 (1.2%)
TP53	7	c.730G>T	p.G244C	Missense	Pathogenic	2 (2.5%)
TP53	7	c.731G>C	p.G244A	Missense	Pathogenic	1 (1.2%)
TP53	7	c.742C>T	p.R248W	Missense	Pathogenic	1 (1.2%)
TP53	7	c.743G>A	p.R248Q	Missense	Pathogenic	3 (3.7%)
TP53	7	c.774A>C	p.E258D	Missense	Pathogenic	1 (1.2%)
TP53	7	c733G>A	p.G245S	Missense	Pathogenic	1 (1.2%)
TP53	8	c..853G>A	p.E285K	Missense	Pathogenic	1 (1.2%)

TP53	8	c.818G>T	p.R273L	Missense	Pathogenic	1 (1.2%)
TP53	8	c.830G>T	p.C277F	Missense	Pathogenic	1 (1.2%)
TP53	9	c.947C>T	p.P316L	Missense	Likely pathogenic	1 (1.2%)
TP53	10	c.1009C>T	p.R337C	Missense	Pathogenic	1 (1.2%)
ZFHX3	2	c.2287delG	p.E763fs*61	Frameshift deletion	Pathogenic	1 (1.2%)

Abbreviations: HGVS, Human Genome Variation Society; ACMG, American College of Medical Genetics and Genomics. Variant type was assigned from the available HGVS nomenclature. Frequency is presented as the number and percentage of patients harboring each unique pathogenic or likely pathogenic variant in the study cohort (n = 81).

Supplementary Table S2. Detailed Characteristics of *POLE* Variants Identified in the Study Cohort

Gene	Exon	HGVS c. Nomenclature	HGVS p. Nomenclature	Classification	POLE Molecular Assignment	No. of Cases
POLE	13	c.1231G>T	p.V411L	Pathogenic	EDM Hotspot	1
POLE	11	c.1082C>T	p.T361I	Likely Pathogenic	EDM	1
POLE	14	c.1394C>T	p.A465V	Likely Pathogenic	EDM	1
POLE	34	c.4337_4338delTG	p.V1446fs*3	Pathogenic	Non-EDM	22
POLE	16	c.1544delA	p.N515Mfs*16	Pathogenic	Non-EDM	1
POLE	4	c.309T>G	p.Y103*	Likely Pathogenic	Non-EDM	1

Total POLE-mutated proxy cases: n = 26

EDM, exonuclease domain mutation; Non-EDM, non-exonuclease domain mutation. EDM hotspot indicates a recognized pathogenic hotspot variant located within the *POLE* exonuclease domain.

Supplementary Table S3. Pathogenic and Likely Pathogenic Variants Identified *in POLE, TP53, BLM, CDC27, MLH3*, and *MSH3* Genes Including Variant Allele Frequency (VAF), Sequencing Coverage, and POLE Molecular Assignment

Case ID	Gene	Exon	HGVS c. Nomenclature	HGVS p. Nomenclature	Classification	VAF (%)	Coverage (×)	POLE Molecular Assignment
**EC001**	*BLM*	7	c.1544delA	p.N515fs*16	Pathogenic	5.52	163	
	TP53	8	c..853G>A	p.E285K	Pathogenic	41	138	
	BLM	7	c.1544delA	p.N515fs*16	Pathogenic	6.01	183	
	MLH3	2	c.1755delA	p.E586fs*24	Pathogenic	4.1	244	
	MSH3	7	c.1148delA	p.K383fs*32	Pathogenic	9.21	391	
	CDC27	3	c.218A>G	p.Y73C	Pathogenic	20	264	
	CDC27	3	c.228A>G	p.A76A	Likely Pathogenic	20	196	
	BLM	7	c.1544delA	p.N515fs*16	Pathogenic	8.24	255	
	MSH3	7	c.1148delA	p.K383fs*32	Pathogenic	5.42	480	
	CDC27	3	c.218A>G	p.Y73C	Pathogenic	18	624	
	CDC27	3	c.204A>C	p.T68T	Pathogenic	20	750	
	CDC27	3	c.228A>G	p.A76A	Likely Pathogenic	17	528	
	CDC27	3	c.218A>G	p.Y73C	Pathogenic	27	148	
	CDC27	3	c.228A>G	p.A76A	Likely Pathogenic	35	233	
	BLM	7	c.1544delA	p.N515fs*16	Pathogenic	4.9	347	
	TP53	6	c.637C>T	p.R213*	Pathogenic	53	203	
	CDC27	7	c.778A>C	p.N260H	Pathogenic	4.88	553	
	CDC27	7	c.761T>G	p.L254*	Pathogenic	6.4	547	
	CDC27	3	c.218A>G	p.Y73C	Pathogenic	4.14	2849	
	CDC27	3	c.228A>G	p.A76A	Likely Pathogenic	4.8	3415	
	CDC27	3	c.204A>C	p.T68T	Likely Pathogenic	6.07	4121	
	TP53	6	c.578A>G	p.H193R	Pathogenic	74	142	
	CDC27	3	c.218A>G	p.Y73C	Pathogenic	5.36	727	
	CDC27	3	c.204A>C	p.T68T	Likely Pathogenic	7.57	898	
**EC002**	*BLM*	7	c.1544delA	p.N515fs*16	Pathogenic	8.68	334	
	MLH3	2	c.1755delA	p.E586fs*24	Pathogenic	4.32	717	
	MSH3	7	c.1148delA	p.K383fs*32	Pathogenic	19	545	
	CDC27	3	c.218A>G	p.Y73C	Pathogenic	22	589	
	CDC27	7	c.228A>G	p.A76A	Likely Pathogenic	19	493	
	CDC27	3	c.204A>C	p.T68T	Likely Pathogenic	25	714	
**EC003**	*BLM*	7	c.1544delA	p.N515fs*16	Pathogenic	7.45	282	
	MLH3	2	c.1755delA	p.E586fs*24	Pathogenic	4.32	556	
	MSH3	7	c.1148delA	p.K383fs*32	Pathogenic	5.65	549	
	CDC27	7	c.778A>C	p.N260H	Pathogenic	16	148	
	CDC27	7	c.761T>G	p.L254*	Pathogenic	19	136	
	CDC27	3	c.218A>G	p.Y73C	Pathogenic	29	529	
	CDC27	3	c.228A>G	p.A76A	Likely Pathogenic	27	441	
	CDC27	3	c.204A>C	p.T68T	Likely Pathogenic	31	686	
	CDC27	3	c.128delT	p.L43fs*19	Likely Pathogenic	4.27	655	
**EC004**	*BLM*	7	c.1544delA	p.N515fs*16	Pathogenic	6.07	676	
	CDC27	3	c.218A>G	p.Y73C	Pathogenic	20	838	

	MSH3	7	c.1148delA	p.K383fs*32	Pathogenic	3.13	702	
	POLE	13	c.1231G>T	p.V411L	Pathogenic	41	274	EDM hotspot
	MLH3	2	c.1755delA	p.E586fs*24	Likely Pathogenic	3.01	1063	
	POLE	4	c.309T>G	p.Y103*	Likely Pathogenic	41	274	Non-EDM
**EC005**	*TP53*	5	c.422G>A	p.C141Y	Pathogenic	9.24	314	
	BLM	7	c.1544delA	p.N515fs*16	Pathogenic	4.73	1523	
	CDC27	7	c.778A>C	p.N260H	Pathogenic	16	1182	
	CDC27	7	c.761T>G	p.L254*	Pathogenic	20	1132	
	CDC27	3	c.218A>G	p.Y73C	Pathogenic	23	2469	
	CDC27	3	c.204A>C	p.T68T	Likely Pathogenic	23	2786	
**EC006**	*TP53*	6	c.578A>G	p.H193R	Pathogenic	63	2550	
	BLM	7	c.1544delA	p.N515fs*16	Pathogenic	6.03	1360	
	MSH3	7	c.1148delA	p.K383fs*32	Pathogenic	2.44	2420	
	CDC27	3	c.218A>G	p.Y73C	Pathogenic	10	1948	
	MLH3	2	c.2021delA	p.N674fs*6	Likely Pathogenic	2.44	2420	
	MLH3	2	c.1755delA	p.E586fs*24	Likely Pathogenic	4.37	2106	
	POLE	34	c.4337_4338delTG	p.V1446fs*3	Pathogenic	2.87	1116	Non-EDM
	BLM	7	c.1544delA	p.N515fs*16	Pathogenic	4.34	1268	
	POLE	34	c.4337_4338delTG	p.V1446fs*3	Pathogenic	2.81	1599	Non-EDM
**EC007**	*BLM*	7	c.1544delA	p.N515fs*16	Pathogenic	4.2	4569	
	MSH3	7	c.1148delA	p.K383FS*32	Pathogenic	3.37	6387	
	MLH3	2	c.1755delA	p.E586fs*24	Pathogenic	3.21	4822	
	TP53	5	c.524G>A	p.R175H	Pathogenic	75	1233	
	CDC27	13	c.1677A>C	p.L559F	Likely Pathogenic	3.95	3949	
	CDC27	3	c.204A>C	p.T68T	Likely Pathogenic	10	9403	
**EC008**	*BLM*	7	c.1544delA	p.N515fs*16	Pathogenic	5.06	1088	
	CDC27	7	c.778A>C	p.N260H	Pathogenic	16	400	
**EC009**	*BLM*	7	c.1544delA	p.N515fs*16	Pathogenic	6.3	1904	
	MSH3	7	c.1148delA	p.K383FS*32	Pathogenic	38	2033	
	CDC27	14	c.1732A>G	p.S578G	Likely Pathogenic	5.17	1817	
**EC0010**	*TP53*	7	c.742C>T	p.R248W	Pathogenic	2.58	426	
	BLM	7	c.1544delA	p.N515fs*16	Pathogenic	5.58	1003	
	CDC27	27	c.761T>G	p.L254*	Pathogenic	20	370	
**EC0011**	*BLM*	7	c.1544delA	p.N515fs*16	Pathogenic	3.27	1130	
	CDC27	3	c.218A>G	p.Y73C	Pathogenic	25	1462	
	CDC27	3	c.228A>G	p.A76A	Likely Pathogenic	25	1378	
	CDC27	3	c.204A>C	p.T68T	Likely Pathogenic	24	1600	
	BLM	7	c.1544delA	p.N515fs*16	Pathogenic	5.36	1512	
	BLM	7	c.1544delA	p.N515fs*16	Pathogenic	4.64	1789	
**EC0012**	*BLM*	7	c.1544delA	p.N515fs*16	Pathogenic	6.16	1348	
	MSH3	7	c.1148delA	p.K383fs*32	Pathogenic	4.22	1919	
	CDC27	7	c.778A>C	p.N260H	Pathogenic	12	566	

	POLE	34	c.4337_4338delTG	p.V1446fs*3	Pathogenic	3.43	1518	Non-EDM
	TP53	7	c.729_730insAGC	p.M243_G244insS	Likely Pathogenic	20	1984	
	CDC27	3	c.228A>G	p.A76A	Likely Pathogenic	8.18	2140	
	BLM	7	c.1544delA	p.N515fs*16	Pathogenic	11	1067	
	MLH3	2	c.1755delA	p.E586fs*24	Pathogenic	4.84	1861	
	CDC27	7	c.778A>C	p.N260H	Pathogenic	2.18	550	
	CDC27	7	c.761T>G	p.L254*	Pathogenic	3.18	534	
	CDC27	3	c.218A>G	p.Y73C	Pathogenic	3.63	1737	
	MSH3	7	c.1148delA	p.K383fs*32	Pathogenic	3.67	1766	
	MLH3	2	c.1043dupT	p.L348fs*13	Likely Pathogenic	5.84	2259	
	POLE	11	c.1082C>T	p.T361I	Likely Pathogenic	2.51	358	EDM
	CDC27	14	c.1905T>G	p.N635K	Likely Pathogenic	5.22	1093	
	CDC27	12	c.1512_1513insC	p.G505fs*6	Likely Pathogenic	4.05	2272	
	CDC27	3	c.228A>G	p.A76A	Likely Pathogenic	4.05	1532	
	CDC27	3	c.204A>C	p.T68T	Likely Pathogenic	3.73	1931	
	TP53	9	c.947C>T	p.P316L	Likely Pathogenic	7.2	236	
**EC0013**	*BLM*	7	c.1544delA	p.N515fs*16	Pathogenic	5.99	167	
	MLH3	2	c.1755delA	p.E586fs*24	Pathogenic	4.21	261	
	MSH3	7	c.1148delA	p.K383fs*32	Pathogenic	9.51	389	
	CDC27	3	c.218A>G	p.Y73C	Pathogenic	42	425	
	CDC27	3	c.228A>G	p.A76A	Likely Pathogenic	40	328	
	CDC27	3	c.204A>C	p.T68T	Likely Pathogenic	48	577	
	CDC27	3	c.128delT	p.L43fs*19	Likely Pathogenic	4.91	346	
**EC0014**	*BLM*	7	c.1544delA	p.N515fs*16	Pathogenic	5.87	477	
	MSH3	7	c.1148delA	p.K383fs*32	Pathogenic	2.41	457	
	TP53	6	c.578A>G	p.H193R	Pathogenic	72	636	
	CDC27	3	c.218A>G	p.Y73C	Pathogenic	28	755	
	MLH3	2	c.1755delA	p.E586fs*24	Likely Pathogenic	3.62	912	
	CDC27	6	c.524G>A	p.N175S	Likely Pathogenic	59	5937	
**EC0015**	*BLM*	7	c.1544delA	p.N515fs*16	Pathogenic	4.26	657	
	CDC27	3	c.218A>G	p.Y73C	Pathogenic	12	933	
	CDC27	7	c.228A>G	p.A76A	Likely Pathogenic	13	872	
	CDC27	3	c.204A>C	p.T68T	Likely Pathogenic	13	976	
**EC0016**	*BLM*	7	c.1544delA	p.N515fs*16	Pathogenic	5.49	1020	
	TP53	5	c.532delC	p.H178fs*69	Pathogenic	2.46	284	
	TP53	5	c.524G>A	p.R175H	Pathogenic	2.78	324	
	CDC27	7	c.778A>C	p.N260H	Pathogenic	7.19	431	

	CDC27	7	c.761T>G	p.L254*	Pathogenic	11	406	
	CDC27	7	c.761T>G	p.L254*	Pathogenic	22	1583	
	CDC27	3	c.218A>G	p.Y73C	Pathogenic	22	1583	
	TP53	4	c.322G>T	p.G108C	Likely Pathogenic	10	501	
	CDC27	3	c.228A>G	p.A76A	Likely Pathogenic	24	1376	
	CDC27	3	c.204A>C	p.T68T	Likely Pathogenic	24	1376	
**EC0017**	*TP53*	5	c.455delC	p.P152fs*18	Pathogenic	32	1516	
	BLM	7	c.1544delA	p.N515fs*16	Pathogenic	6.21	789	
	MSH3	7	c.1148delA	p.K383FS*32	Pathogenic	4.85	1092	
	CDC27	7	c.778A>C	p.N260H	Pathogenic	2.81	285	
	CDC27	7	c.761T>G	p.L254*	Pathogenic	2.68	261	
	CDC27	3	c.218A>G	p.Y73C	Pathogenic	7.16	992	
	BLM	3	c.520delC	p.Q174fs*5	Likely Pathogenic	6.21	789	
	MLH3	2	c.1755delA	p.E586fs*24	Likely Pathogenic	3.19	1443	
	POLE	34	c.4337_4338delTG	p.V1446fs*3	Pathogenic	2.85	1264	Non-EDM
	CDC27	12	c.1515_1516insC	p.G505fs*6	Likely Pathogenic	10	1033	
	CDC27	3	c.228A>G	p.A76A	Likely Pathogenic	7.7	857	
	CDC27	3	c.204A>C	p.T68T	Likely Pathogenic	6.54	1147	
**EC0018**	*BLM*	7	c.1544delA	p.N515fs*16	Pathogenic	5.18	560	
	MLH3	2	c.1755delA	p.E586fs*24	Pathogenic	4.46	897	
	MSH3	7	c.1148delA	p.K383FS*32	Pathogenic	2.36	467	
	POLE	34	c.4337_4338delTG	p.V1446fs*3	Pathogenic	4.1	561	Non-EDM
	TP53	6	c.662_665dupAGCC	p.p223fs*3	Pathogenic	44	298	
	BLM	7	c.1544delA	p.N515fs*16	Pathogenic	5.15	582	
	MLH3	2	c.1755delA	p.E586fs*24	Pathogenic	4.66	687	
	MSH3	7	c.1148delA	p.K383fs*32	Pathogenic	4.35	621	
	POLE	34	c.4337_4338delTG	p.V1446Gfs*3	Pathogenic	2.75	728	Non-EDM
	MLH3	2	c.1755del	p.E586Nfs*24	Pathogenic	3.44	320	
	MSH3	7	c.1148del	p.K383Rfs*32	Pathogenic	2.73	293	
	POLE	34	c.4337_4338del	p.V1446Gfs*3	Pathogenic	2.22	541	Non-EDM
	MLH3	2	c.2021del	p.N674fs*6	Likely Pathogenic	2.32	345	
	BLM	7	c.1544del	p.N515Mfs*16	Pathogenic	5.63	1473	
	POLE	34	c.4337_4338del	p.V1446Gfs*3	Pathogenic	2.82	355	Non-EDM
	POLE	8	c.968dup	p.N323Kfs*2	Pathogenic	2.82	355	Non-EDM
	BLM	7	C.1544del	p.N515Mfs*16	Pathogenic	36	477	
	MLH3	2	c.1755del	p.E586Nfs*24	Pathogenic	3.14	764	
	MSH3	7	c.1148del	p.K383Rfs*32	Pathogenic	23	457	
	POLE	34	c.4337_4338del	p.V1446Gfs*3	Pathogenic	2.18	459	Non-EDM
**EC0019**	*BLM*	7	c.1544del	p.N515Mfs*16	Pathogenic	4.53	486	
**EC0020**	*POLE*	34	c.4337_4338del	p.V144Gfs*3	Pathogenic	3	601	Non-EDM
	TP53	5	c.548C>G	p.S183*	Pathogenic	6.88	189	
	TP53	5	p.423C>G	p.C141W	Pathogenic	4.17	168	
	BLM	7	p.1544del	p.N515Mfs*16	Pathogenic	4.4	159	

	TP53	7	c.730G>T	p.G244C	Pathogenic	34	242	
	BLM	7	c.1544del	p.N515Mfs*16	Pathogenic	4.08	932	
	CDC27	3	c.218A>G	p.Y73C	Pathogenic			
	TP53	7	c.730G>T	p.G244C	Pathogenic	34	242	
**EC0021**	*POLE*	34	c.4337_4338del	p.V1446Gfs*3	Pathogenic	1.18	677	Non-EDM
	BLM	7	c.1544del	p.N515Mfs*16	Pathogenic	4.22	521	
	MLH3	2	c.1755del	p.E586Nfs*24	Pathogenic	2.91	757	
	MSH3	7	c.1148del	p.K383Rfs*32	Pathogenic	2.79	610	
	BLM	7	c.1544del	p.N515Mfs*16	Pathogenic	5.25	1028	
	MLH3	2	c.1755del	p.E586Nfs*24	Pathogenic	4.76	1807	
	POLE	34	c.4337_4338del	p.V1446Gfs*3	Pathogenic	4.15	651	Non-EDM
	TP53	8	c.818G>T	p.R273L	Pathogenic	43	775	
	BLM	7	c.1544del	p.N515Mfs*16	Pathogenic	5.74	575	
	MLH3	2	c.1755del	p.E586Nfs*24	Likely Pathogenic	4.48	1160	
	MSH3	7	c.1148del	p.K383Rfs*32	Pathogenic	4.59	653	
	CDC27	12	c.1515_1516insC	p.G506Rfs*6	Likely Pathogenic	11	700	
	CDC27	7	c.761T>G	p.L254*	Pathogenic	4.08	196	
	CDC27	3	c.228A>G	p.A76A	Likely Pathogenic	19	549	
	CDC27	3	c.218A>G	p.Y73C	Pathogenic	17	667	
	CDC27	3	c.204A>C	p.T68T	Likely Pathogenic	17	763	
**EC0022**	*MLH3*	2	c.1755del	p.E586Nfs*24	Pathogenic	6.83	659	
	POLE	34	c.4337_4338del	p.V1446Gfs*3	Pathogenic	4.01	349	Non-EDM
**EC0023**	*POLE*	34	c.4337_4338del	p.V1446Gfs*3	Pathogenic	4.22	876	Non-EDM
	TP53	7	c.731G>C	p.G244A	Pathogenic	9.52	399	
	BLM	7	c.1544del	p.N515Mfs*16	Pathogenic	3.46	260	
**EC0024**	*BLM*	7	c.1544del	p.N515Mfs*16	Pathogenic	22	2061	
	MLH3	2	c.1755del	p.E586Nfs*24	Likely Pathogenic	6.08	1513	
	MSH3	7	c.1148del	p.K383Rfs*32	Pathogenic	4.43	2550	
	CDC27	7	c.778A>C	p.N260H	Pathogenic	4.15	1832	
	CDC27	7	c.761T>G	p.L254*	Pathogenic	41	8088	
	CDC27	6	c.524A>G	p.N175S	Likely Pathogenic	9.62	2369	
	CDC27	12	c.1515_1516insC	p.G506Rfs*6	Likely Pathogenic	16	233	
	CDC27	14	c.1777G>A	p.A593T	Likely Pathogenic	16	254	
	CDC27	14	c.1908T>G	p.N636K	Likely Pathogenic	10	4342	
**EC0025**	*BLM*	7	c.1544del	p.N515Mfs*16	Pathogenic	5.96	1057	
	MLH3	2	c.1755del	p.E586Nfs*24	Pathogenic	5.37	1768	
	POLE	34	c.4337_4338del	p.V1446Gfs*3	Pathogenic	3.79	581	Non-EDM
	CDC27	7	c.778A>C	p.N260H	Pathogenic	24	518	
	CDC27	7	c.761T>G	p.L254*	Pathogenic	27	465	
	CDC27	3	c.228A>G	p.A76A	Likely Pathogenic	39	1831	
	CDC27	7	c.218A>G	p.Y73C	Pathogenic	35	2179	
**EC0026**	*BLM*	7	c.1544del	p.N515Mfs*16	Pathogenic	5.53	1049	

	MLH3	2	c.1755del	p.E586Nfs*24	Pathogenic	4.12	2328	
	MSH3	7	c.1148del	p.K383Rfs*32	Pathogenic	13	688	
	POLE	34	c.4337_4338del	p.V1446Gfs*3	Pathogenic	3.7	3652	Non-EDM
	CDC27	14	c.1908T>G	p.N636K	Likely Pathogenic	6.8	515	
**EC0027**	*BLM*	7	c.1544del	p.N515Mfs*16	Pathogenic	4.95	2546	
	MLH3	2	c.1755del	p.E586Nfs*24	Pathogenic	4.2	3168	
	CDC27	12	c.1513dup	p.I505Nfs*7	Likely Pathogenic	7.01	7527	
	CDC27	3	c.228A>G	p.A76A	Likely Pathogenic	11	2629	
	CDC27	3	c.218A>G	p.Y73C	Pathogenic	10	2841	
	CDC27	3	c.204A>C	p.T68T	Likely Pathogenic	10	3056	
**EC0028**	*BLM*	7	c.1544del	p.N515Mfs*16	Pathogenic	5.69	2497	
	MLH3	2	c.1755del	p.E586Nfs*24	Pathogenic	4.28	4228	
	MSH3	7	c.1148del	p.K383Rfs*32	Pathogenic	3.78	2193	
	POLE	34	c.4337_4338del	p.V1446Gfs*3	Pathogenic	3.47	2246	Non-EDM
	TP53	7	c.743G>A	p.R248Q	Pathogenic	21	2055	
	CDC27	3	c.228A>G	p.A76A	Pathogenic	20	2296	
	CDC27	3	c.218A>G	p.Y73C	Pathogenic	19	2584	
	CDC27	3	c.204A>C	p.T68T	Likely Pathogenic	18	2924	
	BLM	7	c.1544del	p.N515Mfs*16	Pathogenic	4.94	2512	
**EC0029**	*TP53*	6	c.637C>T	p.R213*	Pathogenic	74	592	
	BLM	7	c.1544del	p.N515Mfs*16	Pathogenic	4.74	2552	
	MLH3	2	c.1755del	p.E586Nfs*24	Pathogenic	3.72	7523	
	CDC27	14	c.1908T>G	p.N636K	Likely Pathogenic	4.39	1231	
	CDC27	13	c.1677A>C	p.L559F	Likely Pathogenic	13	8216	
	CDC27	12	c.1513dup	p.I505Nfs*7	Likely Pathogenic	12	1586	
	CDC27	3	c.228A>G	p.A76A	Likely Pathogenic	12	1735	
	CDC27	3	c.218A>G	p.Y73C	Pathogenic	12	1922	
	CDC27	3	c.204A>C	p.T68T	Likely Pathogenic	12	1735	
	TP53	7	c.683_686del	p.D228Vfs*18	Pathogenic	23	1013	
	TP53	5	c.395A>G	p.K132R	Pathogenic	27	313	
**EC0030**	*POLE*	34	c.4337_4338del	p.V1446Gfs*3	Pathogenic	2.52	953	Non-EDM
	TP53	8	c.830G>T	p.C277F	Pathogenic	33	897	
	BLM	7	c.1544del	p.N515Mfs*16	Pathogenic	4.63	627	
	MSH3	7	c.1148del	p.N515Mfs*16	Pathogenic	5.14	292	
	CDC27	2	c.60T>C	p.A20A	Likely Pathogenic	4.2	405	
**EC0031**	*TP53*	5	c.535del	p.H179Mfs*68	Pathogenic	30	766	
	BLM	7	c.1544del	p.N515Mfs*16	Pathogenic	5.19	1755	
	MSH3	7	c.1148del	p.K383Rfs*32	Pathogenic	3.78	1006	
	CDC27	3	c.228A>G	p.A76A	Likely Pathogenic	10	1989	
	CDC27	3	c.218A>G	p.Y73C	Pathogenic	10	2177	

	CDC27	3	c.204A>C	p.T68T	Likely Pathogenic	9.88	2339	
	BLM	7	c.1544del	p.N515Mfs*16	Pathogenic	4.87	1797	
**EC0032**	*BLM*	7	c.1544del	p.N515Mfs*16	Pathogenic	4.45	2699	
	MLH3	2	c.2021del	p.N674Ifs*6	Likely Pathogenic	16	4923	
	MLH3	2	c.1755del	p.E586Nfs*24	Pathogenic	4.26	4438	
	CDC27	12	c.1515_1516insC	p.G506Rfs*6	Likely Pathogenic	25	8296	
	CDC27	3	c.228A>G	p.A76A	Likely Pathogenic	9.16	2946	
	CDC27	3	c.218A>G	p.Y73C	Pathogenic	8.73	3184	
	CDC27	3	c.204A>C	p.T68T	Likely Pathogenic	8.49	3464	
**EC0033**	*BLM*	7	c.1544del	p.N515Mfs*16	Pathogenic	7.58	501	
	MLH3	2	c.1755del	p.E586Nfs*24	Pathogenic	5.57	467	
	POLE	34	c.4337_4338del	p.V1446Gfs*3	Pathogenic	4.05	791	Non-EDM
	POLE	14	c.1394C>T	p.A465V	Likely Pathogenic	30	1541	EDM
	CDC27	12	c.1515_1516insC	p.G506Rfs*6	Likely Pathogenic	13	565	
	CDC27	3	c.228A>G	p.A76A	Likely Pathogenic	7.74	517	
	CDC27	3	c.218A>G	p.Y73C	Pathogenic	6.17	810	
	CDC27	3	c.204A>C	p.T68T	Likely Pathogenic	7.74	517	
**EC0034**	*BLM*	7	c.1544del	p.N515Mfs*16	Pathogenic	15	2046	
	MLH3	2	c.1755del	p.E586Nfs*24	Pathogenic	4.84	1571	
	MSH3	7	c.1148del	p.K383Rfs*32	Pathogenic	11	2637	
	TP53	4	c.216del	p.V73Wfs*50	Pathogenic	6.78	1180	
	CDC27	7	c.778A>C	p.N260H	Pathogenic	16	246	
	CDC27	7	c.761T>G	p.L254*	Pathogenic	16	283	
**EC0035**	*TP53*	7	c.743G>A	p.R248Q	Pathogenic	41	679	
	BLM	7	c.1544del	p.N515Mfs*16	Pathogenic	5.35	1010	
**EC0036**	*BLM*	7	c.1544del	p.N515Mfs*16	Pathogenic	6.1	2770	
	MLH3	2	c.2021del	p.N674Ifs*6	Pathogenic	2.41	3112	
	MLH3	2	c.1755del	p.E586Nfs*24	Pathogenic	3.85	2729	
	MSH3	7	c.1148del	p.K383Rfs*32	Pathogenic	3.45	3157	
	CDC27	7	c.778A>C	p.N260H	Pathogenic	19	706	
	CDC27	7	c.761T>G	p.L254*	Pathogenic	17	679	
	BLM	7	c.1544del	p.N515Mfs*16	Pathogenic	4.15	1878	
	CDC27	3	c.204A>C	p.T68T	Likely Pathogenic	5.26	209	
**EC0037**	*BLM*	7	c.1544del	p.N515Mfs*16	Pathogenic	5.27	2905	
	MLH3	2	c.1755del	p.E586Nfs*24	Likely Pathogenic	3.78	1271	
	MSH3	7	c.1148del	p.K383Rfs*32	Pathogenic	5.16	2211	
	CDC27	14	c.1908T>G	p.N636K	Likely Pathogenic	4.17	1726	
	CDC27	7	c.778A>C	p.N260H	Pathogenic	14	341	
	CDC27	7	c.761T>G	p.L254*	Pathogenic	15	379	
**EC0038**	*BLM*	7	c.1544del	p.N515Mfs*16	Pathogenic	6.21	1159	

	MLH3	6	c.3571-1G>T		Likely Pathogenic	57	412	
	CDC27	7	c.778A>C	p.N260H	Pathogenic	6.47	340	
	CDC27	7	c.761T>G	p.L254*	Pathogenic	7.72	285	
	CDC27	3	c.228A>G	p.A76A	Likely Pathogenic	14	1011	
	CDC27	3	c.218A>G	p.Y73C	Pathogenic	12	1274	
	CDC27	3	c.204A>C	p.NT68T	Likely Pathogenic	12	1530	
**EC0039**	*BLM*	7	c.1544del	p.N515Mfs*16	Pathogenic	5.61	2139	
	MLH3	2	c.1755del	p.E586Nfs*24	Pathogenic	5.3	3021	
	MSH3	7	c.1148del	p.K383Rfs*32	Pathogenic	4.34	2074	
	CDC27	12	c.1515_1516insC	p.G506Rfs*6	Likely Pathogenic	5.24	4881	
	CDC27	3	c.228A>G	p.A76A	Likely Pathogenic	17	1837	
	CDC27	3	c.218A>G	p.Y73C	Pathogenic	15	2145	
	CDC27	3	c.204A>C	p.T68T	Likely Pathogenic	14	2452	
**EC0040**	*TP53*	7	c733G>A	p.G245S	Pathogenic	13	252	
	BLM	7	c.1544del	p.N515Mfs*16	Pathogenic	7.59	382	
	MLH3	2	c.1755del	p.E586Nfs*24	Pathogenic	5.25	362	
**EC0041**	*BLM*	7	c.1544del	p.N515Mfs*16	Pathogenic	4.75	779	
	MLH3	2	c.1755del	p.E586Nfs*24	Pathogenic	3.07	944	
	MSH3	7	c.1148del	p.K383Rfs*32	Pathogenic	2.57	778	
	POLE	34	c.4337_4338del	p.V1446Gfs*3	Pathogenic	3.47	691	Non-EDM
	TP53	5	c.516del	p.V173*	Pathogenic	3.17	442	
	CDC27	13	c.1677A>C	p.L559F	Likely Pathogenic	13	1121	
	CDC27	13	c.1513dup	p.I505Nfs*7	Likely Pathogenic	12	17468	
	CDC27	3	c.218A>G	p.Y73C	Pathogenic	63	2122	
	CDC27	3	c.204A>C	p.T68T	Likely Pathogenic	61	2346	
	CDC27	1	c.17A>G	p.E6G	Likely Pathogenic	7.93	479	
	CDC27	1	c.4A>G	p.T2A	Likely Pathogenic	9.79	582	
**EC0042**	*POLE*	34	c.4337_4338del	p.V1446Gfs*3	Pathogenic	3.63	937	Non-EDM
	TP53	4	c.318C>G	p.S106R	Pathogenic	84	1539	
	MSH3	7	c.1148del	p.K383Rfs*32	Pathogenic	3.43	700	
**EC0043**	*TP53*	7	c.743G>A	p.R248Q	Pathogenic	9	622	
	BLM	7	c.1544del	p.N515Mfs*16	Pathogenic	5.46	476	
	CDC27	7	c.778A>C	p.N260H	Pathogenic	47	179	
	CDC27	7	c.761T>G	p.L254*	Pathogenic	49	173	
**EC0044**	*POLE*	34	c.4337_4338del	p.V1446Gfs*3	Pathogenic	4.15	1085	Non-EDM
	BLM	7	c.1544del	p.N515Mfs*16	Pathogenic	5.67	952	
	MSH3	7	c.1148del	p.K383Rfs*32	Pathogenic	5.07	1281	
	CDC27	3	c.228A>G	p.A76A	Likely Pathogenic	15	1007	
	CDC27	3	c.218A>G	p.Y73C	Pathogenic	14	1082	
	CDC27	3	c.204A>C	p.T68T	Likely Pathogenic	14	1169	

**EC0045**	*BLM*	7	c.1544del	p.N515Mfs*16	Pathogenic	5.33	1633
	CDC27	3	c.228A>G	p.A76A	Likely Pathogenic	12	1404
	CDC27	3	c.218A>G	p.Y73C	Pathogenic	12	1506
	CDC27	3	c.204A>C	p.T68T	Likely Pathogenic	11	1590
**EC0046**	*BLM*	7	c.1544del	p.N515Mfs*16	Pathogenic	5.18	1507
	MSH3	7	c.1148del	p.K383Rfs*32	Pathogenic	5.7	1035
	TP53	10	c.1009C>T	p.R337C	Pathogenic	32	1625
	TP53	4	c.375+1G>A		Pathogenic	29	1164
	CDC27	12	c.1513dup	p.I505Nfs*7	Likely Pathogenic	5.01	12523
	CDC27	3	c.228A>G	p.A76A	Likely Pathogenic	24	1860
	CDC27	3	c.218A>G	p.Y73C	Pathogenic	24	1971
	CDC27	3	c.204A>C	p.T68T	Likely Pathogenic	24	2094
	BLM	7	c.1544del	p.N515Mfs*16	Pathogenic	4.39	1502
**EC0047**	*BLM*	7	c.1544del	p.N515Mfs*16	Pathogenic	4.86	2183
	MLH3	2	c.1755del	p.E586Nfs*24	Pathogenic	4.5	2179
	MSH3	7	c.1148del	p.K383Rfs*32	Pathogenic	4.99	2845
	CDC27	7	c.778A>C	p.N260H	Pathogenic	12	412
	CDC27	7	c.761T>G	p.L254*	Pathogenic	12	453
**EC0048**	*TP53*	7	c.730G>T	p.G244C	Pathogenic	82	735
	BLM	7	c.1544del	p.N515Mfs*16	Pathogenic	4.57	1729
	MSH3	7	c.1148del	p.K383Rfs*32	Pathogenic	3.89	1953
	CDC27	12	c.1515_1516insC	p.G506Rfs*6	Likely Pathogenic	17	4566
	CDC27	3	c.218A>G	p.Y73C	Pathogenic	13	2214
	CDC27	3	c.204A>C	p.T68T	Likely Pathogenic	12	2547
**EC0049**	*BLM*	7	c.1544del	p.N515Mfs*16	Pathogenic	3.72	1130
	MLH3	2	c.1755del	p.E586Nfs*24	Pathogenic	4.73	1838
	CDC27	12	c.1515_1516insC	p.G506Rfs*6	Likely Pathogenic	34	6579
	CDC27	3	c.228A>G	p.A76A	Likely Pathogenic	19	1391
	CDC27	3	c.218A>G	p.Y73C	Pathogenic	18	1541
	CDC27	3	c.204A>C	p.T68T	Likely Pathogenic	18	1720
**EC0050**	*BLM*	7	c.1544del	p.N515Mfs*16	Pathogenic	6.16	2177
	MLH3	2	c.1755del	p.E586Nfs*24	Pathogenic	4.48	3189
	MSH3	7	c.1148del	p.K383Rfs*32	Pathogenic	3.56	2248
	TP53	7	c.774A>C	p.E258D	Pathogenic	51	778
	CDC27	3	c.218A>G	p.Y73C	Pathogenic	8.21	2667
	MLH3	2	c.1755del	p.E586Nfs*24	Pathogenic	20	2089
	MSH3	3	c.408del	p.K136Nfs*2	Likely Pathogenic	7.35	1375
	MSH3	7	c.1148del	p.K383Rfs*32	Pathogenic	24	1569
	CDC27	3	c.2726_2727del	p.E909Afs*17	Pathogenic	13	1848

VAF, variant allele frequency; EDM, exonuclease domain mutation.

Supplementary Table S4. Overlap of Molecular Classifiers Before Hierarchical TCGA-Inspired Classification

**Combination**	**n**
POLE only	9
MMRd only	7
TP53 only	17
POLE + *TP53*	8
POLE + MMRd	6
MMRd + TP53	3
POLE + MMRd + *TP53*	3
NSMP	28

Counts represent overlapping molecular features identified before application of the hierarchical classification algorithm (*POLE* → MMRd/MSI-H → *p53*-abnormal → NSMP). Therefore, these values do not correspond to the final molecular subgroup assignments reported in Table 5.

Supplementary Table S5. Variant Burden According to TCGA-Inspired Molecular Classification Subgroups

**Molecular Subgroup**	**n**	**Mean Variant Burden ± SD**
POLE-mutated proxy	26	12.85 ± 7.14
MMRd/MSI-H	9	9.6 ± 4.1
p53-abnormal proxy	19	7.4 ± 2.0
NSMP proxy	27	7.1 ± 4.7
